# Integration of Large-Scale Genomic Data Sources With Evolutionary History Reveals Novel Genetic Loci for Congenital Heart Disease

**DOI:** 10.1161/CIRCGEN.119.002694

**Published:** 2019-10-15

**Authors:** Elisavet Fotiou, Simon Williams, Alexandra Martin-Geary, David L. Robertson, Gennadiy Tenin, Kathryn E. Hentges, Bernard Keavney

**Affiliations:** 1 Division of Cardiovascular Sciences, School of Medical Sciences, Faculty of Biology, Medicine, and Health, Manchester Academic Health Science Centre (E.F., S.W., G.T., B.K.), University of Manchester.; 2 Division of Evolution and Genomic science (A.M.-G., D.L.R., K.E.H.), University of Manchester.; 3 MRC-University of Glasgow Centre for Virus Research (D.L.R.).; 4 Manchester Heart Centre, Manchester University NHS Foundation Trust, Manchester (B.K.).

**Keywords:** copy number variants, DNA, evolution, heart defects, congenital, whole exome sequencing

## Abstract

Most cases of congenital heart disease (CHD) are sporadic and nonsyndromic, with poorly understood etiology. Rare genetic variants have been found to affect the risk of sporadic, nonsyndromic CHD, but individual studies to date are of only moderate sizes, and none to date has incorporated the ohnolog status of candidate genes in the analysis. Ohnologs are genes retained from ancestral whole-genome duplications during evolution; multiple lines of evidence suggest ohnologs are overrepresented among dosage-sensitive genes. We integrated large-scale data on rare variants with evolutionary information on ohnolog status to identify novel genetic loci predisposing to CHD.

**Methods:**

We compared copy number variants present in 4634 nonsyndromic CHD cases derived from publicly available data resources and the literature, and >27 000 healthy individuals. We analyzed deletions and duplications independently and identified copy number variant regions exclusive to cases. These data were integrated with whole-exome sequencing data from 829 sporadic, nonsyndromic patients with Tetralogy of Fallot. We placed our findings in an evolutionary context by comparing the proportion of vertebrate ohnologs in CHD cases and controls.

**Results:**

Novel genetic loci in CHD cases were significantly enriched for ohnologs compared with the genome (χ^2^ test, *P*<0.0001, OR =1.253 [95% CI, 1.199–1.309]). We identified 54 novel candidate protein-coding genes supported by both: (1) copy number variant and whole-exome sequencing data; and (2) ohnolog status.

**Conclusions:**

We have identified new CHD candidate loci, and show for the first time that ohnologs are overrepresented among CHD genes. Incorporation of evolutionary metrics may be useful in refining candidate genes emerging from large-scale genetic evaluations of CHD.

## Abstract 2

Supplemental Digital Content is available in the text.

Congenital heart disease (CHD) is the most prevalent birth defect in humans, occurring in ≈8 per 1000 live births, and consisting of malformation of the heart and the great vessels.^1^ Around 20% of all CHDs can be attributed to chromosomal imbalances such as Down and Turner, and 22q11 deletion syndromes; around 80% occur as sporadic, nonsyndromic CHD. In such cases, CHD behaves overall as a genetically complex trait with moderate heritability. Previous genome-wide investigations into CHD have found evidence for rare causative copy number variants (CNVs) and single nucleotide variants (SNVs), and associations with common SNVs in GWAS.^2–6^ It has been estimated in previous studies that several hundred genes may be involved in polygenic CHD susceptibility; therefore, many remain to be discovered.^7^

CNVs are 1 kilobase (kb) to several megabase (Mb) sized regions of duplication and deletion in the genome. A 2014 meta-analysis of CNVs in 1694 nonsyndromic CHD cases identified 79 chromosomal regions in which 5 or more CHD cases had overlapping imbalances.^5^ The estimated prevalence of pathogenic CNVs in nonsyndromic CHD patients is 4% to 14%, whereas in syndromic CHD patients it is 15% to 20% (the most common being 22q11 deletion syndrome).^3,8,9^ There are multiple mechanisms by which a CNV may lead to disease including the disruption of chromosome structure, alteration of gene expression due to disruption of regulatory elements, and changes of the relative amounts of dosage-sensitive genes.^10^

The dosage-balance model postulates that, for genes that are in stoichiometric relationships (eg, forming protein complexes with other genes), any perturbation in their relative ratios will tend to be deleterious.^10^ In the early course of vertebrate evolution, around 500 million years ago, 2 whole-genome duplications, during which gene stoichiometry throughout the genome was preserved, as all genes were duplicated, took place. Periods of gene loss followed each of these events, resulting in the retention of some whole-genome duplication paralogs in the genome (termed ohnologs) and the loss of others. The dosage-balance model would predict that ohnologs should be enriched for dosage-sensitive genes.^11^ Ohnologs, of which there are around 7000 in the human genome, have indeed been shown to exhibit characteristics consistent with dosage-sensitivity: for example, ohnologs are enriched for haploinsufficient genes^11,12^; and Makino et al^13^ reported, based on CNV data in healthy individuals from the Database of Genomic Variants, that genomic regions (≈2 Mb in size) near ohnologs are CNV deserts, indicating that those regions are dosage-balanced.

The formation and fixation of gene duplications within the genome are subject to different evolutionary mechanisms–small scale duplications (SSD) involving relatively few genes, and whole-genome duplication. A strong relationship between the evolutionary mechanism of duplication and phenotypic consequences, including heritable diseases, has been previously shown.^14–16^ Ohnologs have a significant association with certain human genetic diseases; for example 12 out of 16 reported candidate genes within the Down syndrome critical region (21q22.12, 21q22.13, and 21q22.2) are dosage-balanced ohnologs.^11^ By contrast, genes arising from SSDs lack enrichment for disease association.^17^ In addition, ohnologs are enriched for genes involved in signaling and gene regulation, key cardiovascular developmental processes.^11^ These considerations led us to hypothesize that ohnologs may be enriched among CHD causative genes.

We tested this hypothesis in a meta-analysis of CNV data including 4634 nonsyndromic CHD cases, and integrated these data with a WES (whole-exome sequencing) study of 829 cases of Tetralogy of Fallot (TOF), the commonest cyanotic CHD phenotype, which has been previously shown to have a significant etiological contribution from CNVs.^6^ These were compared with control data, which were derived from large-scale genomic resources.^18–21^

## Methods

The appropriate institutional review bodies approved all recruitment of human participants in this study. The study corresponded with the stipulations of the Declaration of Helsinki, and all participants (or their parents, if affected probands were children too young to themselves consent) provided informed consent. Data from consortia were accessed subject to the applicable data-sharing agreements. Summary data, analytic methods, and summary study materials will be made available to other researchers for purposes of reproducing the results or replicating the analyses reported here, on request to the corresponding authors. Full Materials and Methods are available in the Data Supplement of the article.

## Results

### Update of CHD CNV Data Set and Generation of Control CNV Data Set

**Figure 1.**
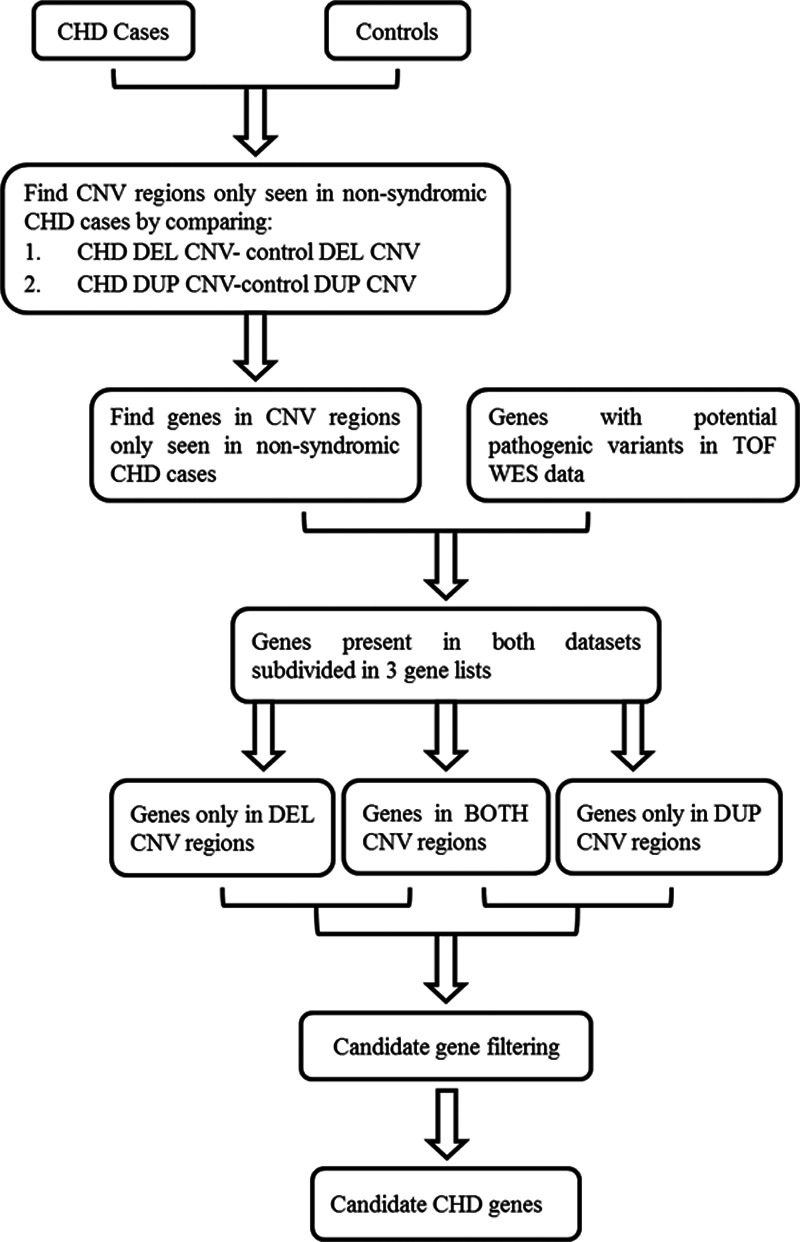
**Overall methodology.** Flowchart showing the methodology used to identify novel genetic loci for nonsyndromic congenital heart disease (CHD) cases. Potential pathogenic variants were novel or rare single nucleotide variants (either absent from ExAC or with frequency of <0.01). Candidate genes identified at the end of the workflow were subsequently analyzed for ohnolog status. BOTH indicates deleted/duplicated; CNV, copy number variant; DEL, deletion; DUP, duplication; TOF, Tetralogy of Fallot; and WES, whole-exome sequencing.

We updated the previous meta-analysis of CNVs in nonsyndromic CHD cases,^5^ in a further 2882 nonsyndromic CHD cases from DECIPHER (Database of Chromosomal Imbalance and Phenotype in Humans using Ensembl Resources), ECARUCA (European Cytogeneticists Association Register of Unbalanced Chromosome Abberations), ISCA (International Standards for Cytogenomic Arrays) databases and further published studies investigating the role of CNVs in CHD.^4,20,22–32^ The updated CHD case CNV data set consists of 4634 unrelated individuals of different ancestries (Table 1). The outline workflow to identify candidate genes is shown in Figure 1. Filtering of the CHD case population against DECIPHER known microdeletion/microduplication syndromes resulted in 224 cases being removed; this left 4410 CHD cases with 3362 deletion CNVs and 2540 duplication CNVs which were used for further analysis (Figure I in the Data Supplement).

**Table 1.**
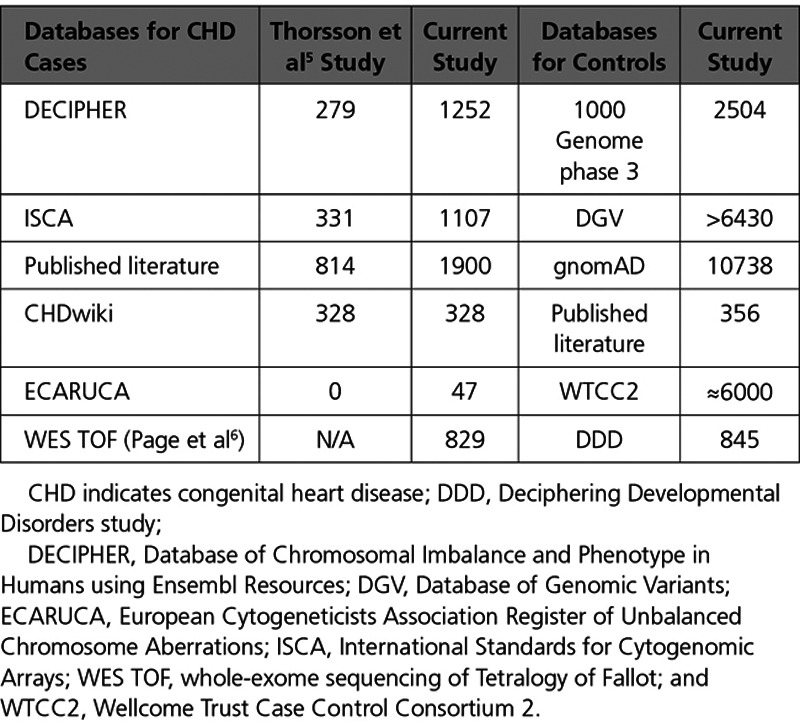
Number of Cases in Previous and Current Meta-Analysis Studies as Well as Controls Used in the Current Study

A control CNV data set was generated by acquiring CNV data from individuals not explicitly identified as having a developmental disorder, who were enrolled in the 1000 Genome Project Phase 3, Database of Genomic Variants, DECIPHER, and published studies.^21,27,28,33,34^ The control CNV data set resulted in 256 511 deletion CNVs, 84 343 duplication CNVs, and 6403 BOTH CNVs, that is, either deletion or duplication. gnomAD CNVs^35^ were incorporated into the analysis as they became available and resulted in an additional 51 420 duplication CNVs and 198 611 deletion CNVs.

### Comparison of CHD CNV Regions With Control CNV Regions

All CHD deletion and duplication CNV regions (coordinates hg19) were compared against control deletion and duplication CNV regions, respectively. Any CHD CNV regions overlapping control CNV regions were excluded. As a result, we identified deletion and duplication CNV regions only seen in nonsyndromic CHD cases. The genes located in those regions were annotated using the Ensembl database. There were a number of genes that already had an assigned phenotype (Online Mendelian Inheritance in Man)^36^; among these, 59 had been previously associated with CHD pathogenesis such as *ZIC3*, *NKX2-6*, *GATA4*, *JAG1*, *GJA1*, and *TBX5*. All genes with Online Mendelian Inheritance in Man assigned phenotypes were excluded from further analysis.

**Figure 2.**
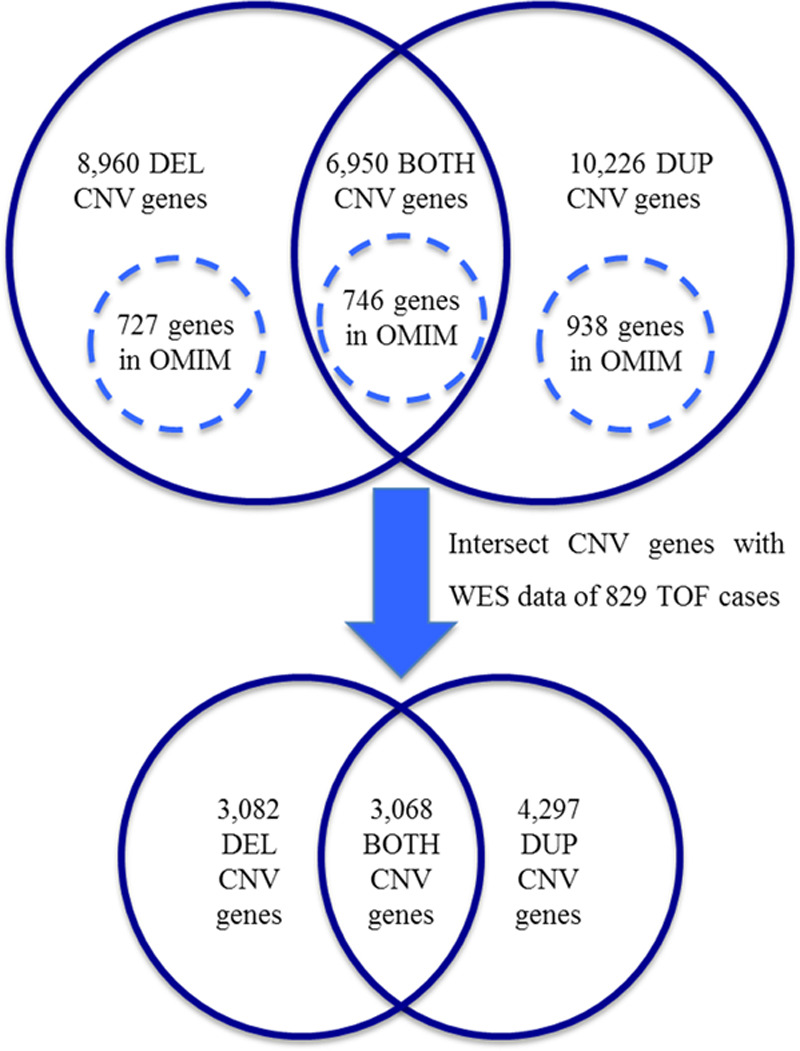
**Intersection of copy number variant (CNV) and whole-exome sequencing (WES) data.** Numbers of genes involved in the final stages of the workflow depicted in Figure 1 are shown. Genes with assigned phenotypes (circles with dashed line) were excluded from further analysis. BOTH indicates deleted/duplicated; DEL, deletion; DUP, duplication; OMIM, Online Mendelian Inheritance in Man; and TOF, Tetralogy of Fallot.

Novel genes found in the CNV regions only seen in CHD cases were then compared with an in-house list of 12 771 genes with novel or rare SNVs (either absent from ExAC or with frequency of <0.01) from WES data in 829 TOF cases.^6^ Genes supported by both CNV and WES data were included for further analysis. In total, 3082 genes in deletion CNVs, 4297 genes in duplication CNVs, and 3068 in BOTH CNVs (ie, genes found in deletion and duplication CNVs) were also found in the TOF WES data with either high (nonsense variants, frameshift, splice variants) or medium (missense, splice variants) impact SNVs. This intersection of CNV and WES data led to an overall reduction of ≈60% in the number of candidate genes for CHD (Figure 2).

### Ohnologs are Highly Enriched in CHD Cases Whereas SSD and Singleton Genes are Not

**Figure 3.**
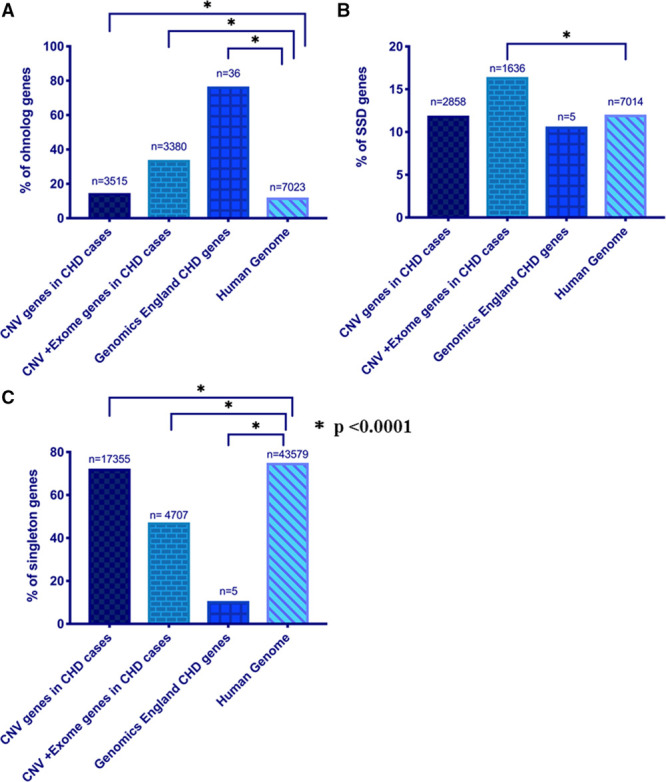
**Ohnologs are enriched in congenital heart disease (CHD) cases.** Graphs show the percentage of genes that are (**A**) ohnologs, (**B**) small scale duplications (SSD), and (**C**) singletons. Statistical significance was tested using 2-tailed χ^2^ test with Yates’s correction, *P*<0.05 was considered statistically significant. CNV indicates copy number variant.

Ohnologs (N=7023) were identified using data from Singh et al,^37^ available at http://ohnologs.curie.fr/. SSDs (N=7014) were extracted from Ensembl gene trees.^12^ Any remaining genes that were neither found in the ohnolog data set nor identified as having a direct paralog were considered for the purpose of this study to be singletons. The frequencies of ohnologs, SSDs, and singletons among the candidate CHD genes were compared with their frequency in the human genome. Novel genes supported by the CNV data in CHD cases were found to be enriched for ohnologs (14.65% versus 12.05%, χ^2^ test, *P*<0.0001; OR=1.253 [95% CI, 1.199–1.309]; Figure 3A). There were no differences in SSDs (Figure 3B) and an under-representation for singletons (Figure 3C) compared with the human genome. There was a 2.3-fold increased enrichment of ohnologs in the genes supported by both CNV and WES data in CHD cases (χ^2^ test, *P*<0.0001, OR=3.751 [95% CI, 3.574–3.937]). In this instance, SSDs were also enriched in CHD cases compared with the human genome (χ^2^ test, *P*<0.0001; OR=1.437 [95% CI, 1.356–1.905]). However, ohnologs were 2-times elevated compared with SSD genes (33.94% versus 16.43%). Additionally, we assessed our methodology by applying it to a group of genes with strong a priori evidence for pathogenicity. The crowd-sourced Genomics England PanelApp gene list for CHD (available at https://panelapp.genomicsengland.co.uk/panels/212/), which represents a consensus view of causative genes, was highly enriched for ohnologs (76.6% versus 12.05%, χ^2^ test, *P*<0.0001; OR=23.89 [95% CI, 12.33–46.18]). We, therefore, used ohnolog status as an additional candidate gene filter.

### Candidate Genes Supported by Both CNV and WES Data of CHD Cases

**Figure 4.**
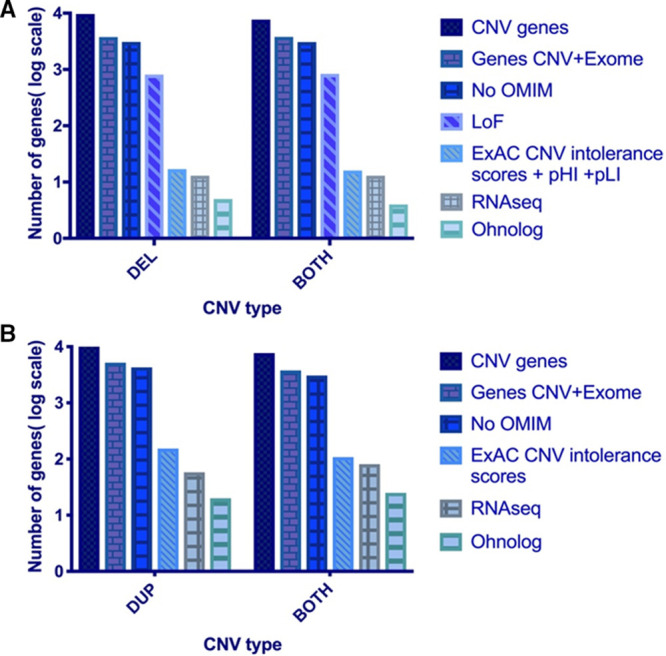
**Filtering process using large-scale genomic data resources.** Both graphs are in logarithmic scale and represent the consecutive filtering of the genes using the different metrics for (**A**) deleted (DEL) and both copy number variant (CNV) genes (**B**) duplicated (DUP) and both CNV genes. There is ≈70% reduction in the number of candidate genes when we apply the evolutionary duplication metric—ohnolog. Also, none of our candidates were present in the list of homozygously deleted genes (nonessential) from the Sudmant study as well as not present in the list of genes curated from the DDD study. LoF indicates loss-of-function; OMIM, Online Mendelian Inheritance in Man; pHI, probability of haploinsuffieciency; and pLI, probability of loss-of-function intolerance.

To further refine our candidate genes, we integrated additional genomic resources including the top 5% ExAC CNV intolerance scores, probability of haploinsuffieciency (pHI),^38^ probability of loss-of-function intolerance (pLI),^19^ and RNAseq expression data from mouse embryonic hearts.^39^ Lastly, we incorporated ohnolog status. Genes from BOTH CNVs were analyzed twice; once with the metrics used for genes from deletion CNVs and once with the metrics used for genes from duplication CNVs (Figure 4).

This led to the identification of 9 candidate genes from deletion and BOTH CNVs: *BRWD1*, *DIP2C*, *EYA3*, *GRB10, HNRNPC, RC3H2, SLIT3, TLN1*, and *UBASH3B*. All 9 have the following properties: (1) loss-of-function (LoF) variants in the WES data, (2) found in deletion or BOTH CNV regions only seen in nonsyndromic CHD cases, (3) top 5% of ExAC deletion CNV intolerance scores, (4) haploinsufficient (pHI ≥0.65) and unable to tolerate loss-of-function variants (pLI ≥0.9), (5) in the top 25% of highly expressed genes in mouse heart at E9.5 and E14.5, (6) ohnolog, (7) not present in the list of genes curated from the DDD study (Deciphering Developmental Disorders), and (8) not classified as human nonessential genes from the Sudmant study^21^ (Table 2).

**Table 2.**
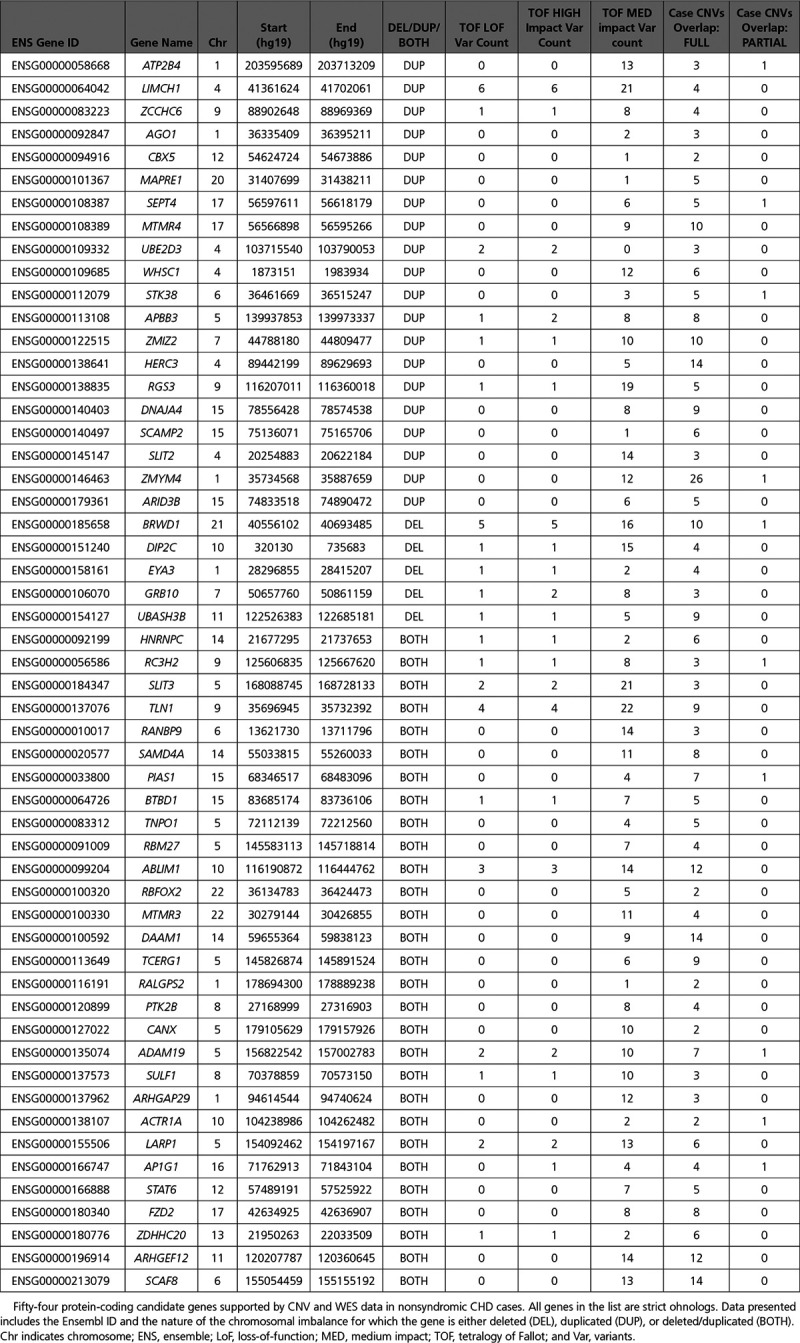
Candidate Genes Supported by both CNV and WES Data of CHD Cases

In addition, we found 45 candidate genes from duplication and BOTH CNVs, which had the following properties: (1) high or medium impact SNVs in the WES data, (2) found in duplication and BOTH CNV regions only seen in nonsyndromic CHD cases, (3) top 5% of ExAC duplication CNV intolerance scores, (4) in the top 25% of highly expressed genes in mouse heart at E9.5 and E14.5, (5) ohnolog, (6) not present in the list of genes curated from the DDD study, and (7) not in the list of nonessential human genes from the Sudmant study^21^ (Table 2).

### Pathway Enrichment and Gene Ontology Analysis

**Figure 5.**
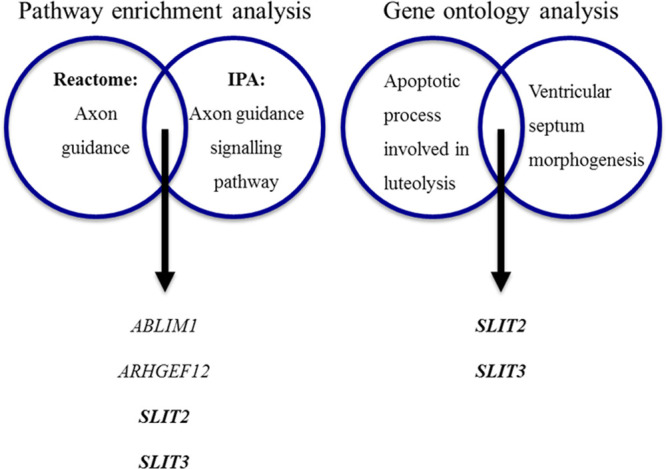
**Genes in the top significant pathways and biological processes.** *SLIT2* and *SLIT3* genes were supported by multiple lines of evidence. IPA indicates ingenuity pathway analysis.

We performed pathway enrichment analysis, using the Reactome Pathways Analysis tool,^40^ on the final 54 candidate genes supported by both CNV and WES data in nonsyndromic CHD cases. This resulted in 11 pathways, where >5 of our candidate genes were involved in those pathways (Table 3). The top 3 pathways based on entities ratio (entities found/total entities) from Reactome were axon guidance, signaling by receptor tyrosine kinases, and cellular responses to external stimuli. In addition, ingenuity pathway analysis was also used with the only pathway including >5 genes being axon guidance signaling. Gene ontology (GO) analysis^41^ of our candidate genes revealed 22 GO terms with particular enrichment on 4 GO terms; apoptotic process involved in luteolysis (GO0061364; FDR corrected *P* value=0.0462), ventricular septum morphogenesis (GO0060412; FDR corrected *P* value=0.00921), ventricular septum development (GO0003281; FDR corrected *P* value=0.0343), and cardiac septum morphogenesis (GO0060411; FDR corrected *P* value=0.036). Both pathway and GO analysis identified processes in which the genes *ABLIM1*, *ARHGEF12*, *SLIT2*, and *SLIT3* are involved (Figure 5).

**Table 3.**
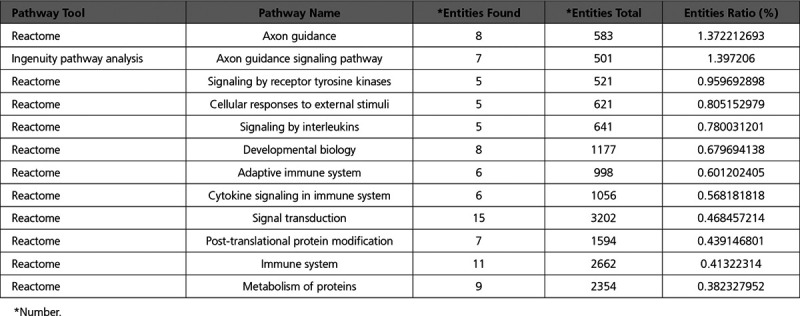
Top Pathways Overrepresented in Our 54 Candidate Genes

### *SLIT2* and *SLIT3* Variants in CHD

*SLIT2* and *SLIT3* were the most strongly supported genes found both by pathway analysis and GO (Figure 5). Therefore, we further explored the phenotypic associations of these genes within our population.

In the present study, individuals with CNVs including *SLIT3* were reported with malformation of the heart and great vessels (n=1), ventricular septal defect (n=1), atrial septal defect (n=3), and TOF (n=1) whereas individuals with *SLIT2* CNVs were reported with malformation of the heart and great vessels (n=1), ventricular septal defect (n=2), and double outlet right ventricle (n=1). In addition, 20 missense SNVs and 3 splice-site SNVs in *SLIT3* were found in 24 out of 829 TOF cases (2.9% [95% CI, 1.91%–4.35%]) and *SLIT2* had 12 missense SNVs and 2 splice-site SNVs in 14 out of 829 TOF cases (1.7% [95% CI, 0.9%–2.9%]). Probands were available for 12 *SLIT3* variants, and 5 *SLIT2* variants which were confirmed by Sanger sequencing. Remaining variants were confirmed to have good coverage using Integrative Genomics Viewer. Samples from both parents were available for 9 probands with *SLIT3* variants and were analyzed for variant inheritance; 2 of the 9 *SLIT3* variants tested were identified as de novo. Samples from both parents were available for 5 probands with *SLIT2* variants and were all either maternally or paternally inherited.

## Discussion

Here, we performed a large-scale genome-wide meta-analysis study of nonsyndromic CHD cases and identified 54 novel candidate genes for CHD. In addition to the large size of our data set, we incorporated a novel analysis strategy incorporating the evolutionary origin of gene duplications. Ohnologs tend not to be observed in CNVs in vertebrate genomes.^13^ Moreover, McLysaght et al^14^ have also shown that ohnologs are significantly overrepresented in pathogenic CNVs associated with schizophrenia and neurodevelopmental disorders and that they are the possible cause of the deleterious effects of these rare pathogenic CNVs. Here, we have shown for the first time that genes included in CNVs from CHD cases are significantly enriched for ohnologs compared with the human genome. Due to this significant association between ohnologs and CHD, we incorporated ohnolog status in our methodology to identify novel genetic loci associated with CHD.

Pathway analysis and GO analysis concordantly identified the *SLIT2* and *SLIT3* genes, which have recently received increasing attention in heart development.^42^ Invertebrates, the Slit family comprises of 3 known members (SLIT1-3), which are highly conserved secreted proteins that bind to ROBO (Roundabout receptors). *SLIT2* and *SLIT3* are expressed during mouse embryonic development and interact with ROBO1 and ROBO2.^43,44^ They both encode proteins that consist of 4 LRR domains (leucine-rich repeats) also called D1-D4, 8 EGF repeats (epidermal growth factor) and 1 Laminin-G-like domain.^43^ *Slit3* is expressed early in murine cardiogenesis in the cardiac crescent at E7.5 and linear heart tube at E8.5, later expression being restricted to the myocardium of the atria and OFT but not in the cardiac cushions or valves.^44,45^ *Slit2* is strongly expressed in the pharyngeal region at E8.5-E9.5 and later in the ventricular trabecular myocardium, epicardium, aortic semilunar valves and the mesenchyme surrounding the caval veins.^44,46^ *Slit3*^−/−^ mutant mice exhibit ventricular septal defect, thick atrioventricular valves, and hypoplastic posterior aortic semilunar leaflet with Slit2^−/−^ mutant mice exhibiting bicuspid aortic valves and immature semilunar valves.^44^ Robo1^−/−^ mutant mice also exhibit ventricular septal defect with downregulation of Notch signaling, suggesting a potential mechanism for the underlying defects.^44^ In another study, *Slit3*^−/−^ mice also exhibit congenital diaphragmatic hernia.^47^ Congenital heart defects ranging from bicuspid aortic valves to septal, and outflow tract defects are, therefore, observed in variety of animal models in which genes involved in the SLIT/ROBO pathway have been inactivated.

We identified *SLIT2* and *SLIT3* heterozygous SNVs in 2.9% and 1.7% nonsyndromic TOF patients, respectively. All SNVs were novel or rare (either absent from ExAC or with frequency of <0.01) and predicted with in silico tools to be pathogenic. The majority of the SNVs in both genes were missense, although a few splice-site SNVs were also found. Their functional relevance will be of interest in future studies. Both genes were also present in CNVs in patients with CHD with varying phenotypes, including septal defects and malformation of the great arteries. This is the first study to find an association of *SLIT2* and *SLIT3* with predisposition to human CHD, although, of note, a recent study identified *ROBO1* loss-of-function SNVs in cases with TOF and septal defects.^48^

### Limitations

This study has certain limitations. The databases and publications included in this analysis incorporated different CNV detection platforms and analysis algorithms.^49^ Irrespective of the method used in the studies identifying pathogenic CNVs, we only included studies that used the same genotyping method between cases and controls and confirmed their CNV detection by an additional methodology like quantitative polymerase chain reaction, which to a degree addresses this limitation. Another potential limitation is the fact that during our filtering strategy, we might have missed some important genes crucial for cardiac development. Though we accept that all important genes will not have been captured, we detected 54 strong candidate genes supported by multiple lines of evidence as having a causative role in nonsyndromic CHD. Further research in much larger numbers of comprehensively genetically characterized CHD cases is warranted to establish the magnitude of the contribution of these genes, and to discover novel loci.

In conclusion, we show that ohnologs are overrepresented in CHD cases and that incorporation of the evolutionary origins of genes is useful in refining candidate genes emerging from large-scale genetic evaluations of CHD. We also observe that CNVs and SNVs in *SLIT2* and *SLIT3* are associated with CHD involving TOF, septal defects, and outflow tract defects, supporting the importance of the SLIT-ROBO signaling pathway in heart development.

## Acknowledgments

This study makes use of data generated by the DECIPHER Consortium. A full list of centers that contributed to the generation of the data is available from https://decipher.sanger.ac.uk/ and via email from decipher@sanger.ac.uk. Funding for the DECIPHER project was provided by the Wellcome Trust.^20^

## Sources of Funding

This study was supported by the British Heart Foundation. A. Martin-Geary is funded by a PhD studentship from the Medical Research Council (number 1622139). Prof Robertson is partially funded by the Medical Research Council (MC UU 1201412). Prof Keavney holds a British Heart Foundation Personal Chair.

## Disclosures

None.

## Supplementary Material
