# Supplementary material for: Integration of Large-Scale Genomic Data Sources With Evolutionary History Reveals Novel Genetic Loci for Congenital Heart Disease
Source: Circ Genom Precis Med. 2019 Oct 15;12(10):e002694. doi: 10.1161/CIRCGEN.119.002694 (PMC6798745; doi:10.1161/CIRCGEN.119.002694)
Supplement: Supplementary file 1 [file hcg-12-e002694-s001.pdf]

SUPPLEMENTAL MATERIAL

Methods

Database search and update of the CHD case CNVs dataset using published data

The complete list of all CNV coordinates (not only the 79 chromosomal regions in which 5 or more CHD patients had overlapping CNVs) from the previous meta-analysis by Thorsson *et al.* was obtained in order to be further updated[1]. We have excluded 8 cases from the list obtained, who had only patent foramen ovale as their phenotype. The following four sources were used to identify further non-syndromic CHD patients: a) The Database of Chromosomal Imbalance and Phenotype in Humans using Ensembl Resources (DECIPHER) database, b) The International Standards for Cytogenomic Arrays (ISCA) database, c) European Cytogeneticists Association Register of Unbalanced Chromosome Aberrations (ECARUCA) database, d) Pubmed search of publications examining CNV association with CHD pathogenesis.

The Database of Chromosomal Imbalance and Phenotype in Humans using Ensembl Resources (DECIPHER)

All necessary agreements were signed from both parties for the use of the data on DECIPHER database[2]. The DECIPHER case CNV list, which includes data from more than 250 academic departments of clinical genetics and rare disease genomics with more than 27,000 cases, was filtered using the terms as shown in supplementary table 1. A list of known rare syndromes associated with CNVs (for example 22q11 deletion, Williams' syndrome) was also obtained from DECIPHER. Comparison of the CHD case CNV list against the DECIPHER CNV-known syndromes was performed. Every CHD case CNV was either not matching any CNV-known

syndrome or was assigned as WITHIN, PARTIAL or FULL with 1 or more CNV-known syndrome/s (Supplementary figure 1). Any CHD case CNVs that were assigned as WITHIN (except the ones within 1q21.1 and 8p23.1 regions previously known to be associated with CHD) were excluded from further analysis. Finally, 871 DEL CNVs and 693 DUP CNVs were identified to be present in 1188 individuals reported with one or more cardiac defects (supplementary table 1) and with or without additional extracardiac abnormalities.

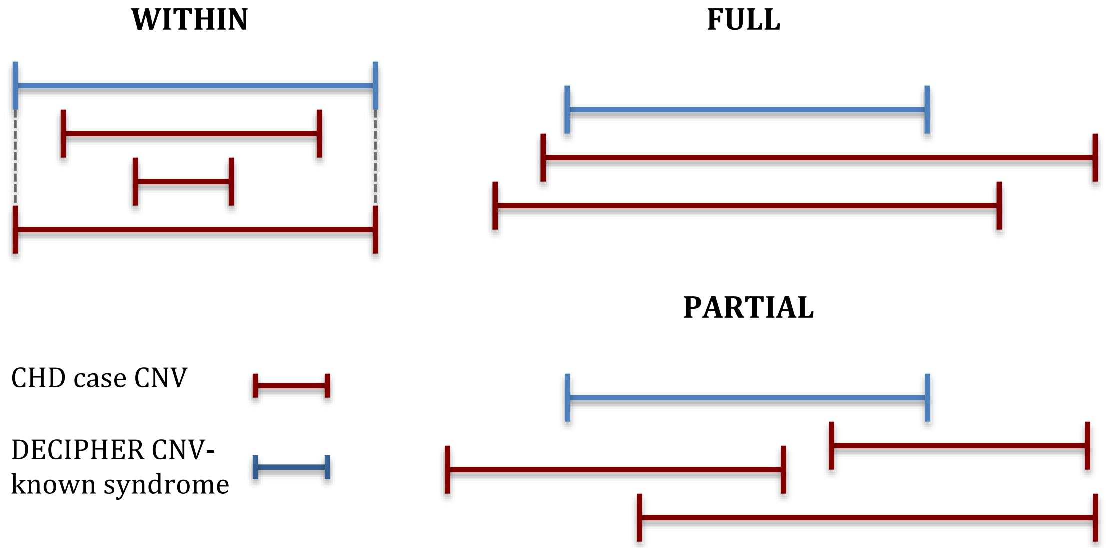

Supplementary figure 1: Schematic diagram showing the comparison of DECIPHER CNV-known syndromes against our CHD case CNVs. If the CHD case CNV falls within the boundaries of the CNV-known syndrome/s or matches exactly, then it was assigned as WITHIN. If the CHD case CNV overlaps either one of the boundaries of the CNV-known syndrome/s, then it was assigned as PARTIAL. If the CHD case CNV fully covers the boundaries of the CNV-known syndrome/s and extends at either side, then it was assigned as FULL.

The International Standards for Cytogenomic Arrays (ISCA) consortium

The ISCA database was accessed through the Clinical Genome (ClinGen) database (http://dbsearch.clinicalgenome.org/search/) and the search terms that were used to find further CHD patients with CNVs can been found in supplementary table 1. Moreover, additional cases from ISCA were identified in dbVar under the studies nstd101 and nstd37[3, 4]. After the exclusion of CHD case CNVs that were WITHIN any CNV-known syndromes, there were 1,045 cases from ISCA with 495 DEL CNVs and 550 DUP CNVs.

European Cytogeneticists Association Register of Unbalanced Chromosome Aberrations (ECARUCA)

A bulk query of patients with heart abnormalities analysed with microarray was requested from ECARUCA[5]. This resulted in 82 cases with heart defects and with or without other abnormalities (supplementary table 1). Cases with abnormal karyotype were excluded from further analysis (10 out of 82 cases). Moreover, another 24 cases were excluded, as there was no information of their karyotype. A single case that had CNV coordinates from the hg17 assembly was not successfully converted to hg19. After the exclusion of any CNVs WHITHIN CNV-known syndromes, this left us with 43 cases with a total of 60 CNVs, for which 34 were DEL CNVs and 18 were DUP CNVs.

Search terms	DECIPHER	ISCA	ECARUCA
**Abnormal atrioventricular connection**	x		
**Abnormal cardiac septum morphology**	x		
**Abnormal cardiac ventricle morphology**	x		
**Abnormal heart morphology**	x	x	
**Abnormality of aortic valve**		x	
**Abnormality of mitral valve/ of the mitral valve**	x	x	
**Abnormality of pulmonary valve**		x	
**Abnormality of right ventricle**		x	
**Abnormality of the aorta**	x		
**Abnormality of the atrial septum**	x		
**Abnormality of the cardiovascular system**	x		
**Abnormality of the vena cava**	x		
**Abnormality of the ventricular septum**	x		
**Abnormality of tricuspid valve**		x	
**Abnormality of vena cava**		x	
**Abnormality of ventricular septum**		x	
**Anomalous pulmonary venous return**	x	x	
**Anomalous venous return**			x
**Aortic aneurysm**		x	
**Aortic incompetence**			x
**Aortic stenosis**	x		x
**Aortic valve atresia**		x	

**Aortic valve stenosis**	x	x	
**Atria septal defect**	x	x	
**Atrial septum defect**			x
**Atrioventricular block**		x	
**Atrioventricular canal defect**	x	x	
**Atrioventricular septal defect**			x
**Bicuspid aortic valve**	x	x	
**Cardiomegaly**	x		
**Coarctation of aorta/of the aorta**	x	x	x
**Congenital cardiac anomaly**			x
**Congenital malformation of the great arteries**	x		
**Conotruncal defect**	x		
**Dextrocardia**	x	x	
**Double outlet right ventricle**	x	x	
**Ebstein’s anomaly**	x		
**Ebstein's anomaly of tricuspid valve**		x	
**Heart**	x	x	
**Heart, general abnormalities**			x
**Hypoplastic left heart**		x	x
**Hypoplastic left heart syndrome**	x		
**Hypoplastic right heart**		x	
**Interrupted aortic arch**	x	x	
**Interrupted/Hypoplastic aorta**			x
**Left anterior mitral valve leaflet**	x		

**Malformation of the heart and great vessels**		x	
**Mitral incompetence**			x
**Mitral regurgitation**	x		
**Mitral stenosis**	x	x	
**Mitral valve prolapse**		x	
**Partial anomalous venous return**		x	
**Patent ductus arteriosus**	x	x	x
**Pulmonary artery atresia**		x	
**Pulmonary artery stenosis**	x		
**Pulmonary atresia**			x
**Pulmonary hypoplasia**		x	
**Pulmonary stenosis**	x		x
**Pulmonic stenosis**	x	x	
**Single ventricle**		x	
**Supravalvular aortic stenosis**		x	
**Tetralogy of Fallot**	x	x	x
**Total anomalous venous return**		x	
**Transposition of great arteries/of the great arteries**	x	x	
**Tricuspid atresia**	x	x	
**Tricuspid incompetence**			x
**Tricuspid regurgitation**	x		
**Tricuspid stenosis**	x		
**Truncus arteriosus**	x	x	

**Ventricular hypertrophy**	x		
**Ventricular septal defect**	x	x	
**Ventricular septum defect**			x

Supplementary Table 1| Search terms for DECIPHER, ISCA and ECARUCA databases: This table shows the search terms that have been used in the different databases to identify CHD cases with reported CNVs. Note some redundancy dependent upon classification schemes used in each database; also "Aortic stenosis" indicates congenital rather than subsequently acquired aortic stenosis.

Published studies

Data were pooled from 9 publications, which examined the association of CNVs with CHD[6-11]. The methods of CNV identification and verification are described in each publication[6-11]. The number of cases as well as the number of DEL and DUP CNVs of each publication that have been included in the CHD case CNV dataset, after the exclusion of any CNVs within CNV-known syndromes, are shown in supplementary table 2.

**Publication**	**Number of cases**	**Number of DEL CNVs included in this study**	**Number of DUP CNVs included in this study**
Fakhro *et al.* (2011)	40	15	28
Hitz *et al.* (2012)	61	32	45
Glessner *et al.* (2014)	51	35	24
Rigler *et al.* (2015)	19	12	12
Hightower *et al.* (2015)	25	11	14
Sanchez-Castro *et al.* (2016)	64	21	49
Xie *et al.* (2014)	14	8	7
Hanchard *et al.* (2017)	30	14	19
Xie *et al.* (2017)	782	1222	538

Supplementary table 2| Copy number variants from published studies: CNVs from the nine publications listed in this table were added in the CHD case CNV dataset. The number of cases is not the total number of cases in each publication but the number of cases that have been used in our study as some cases were excluded from further analysis due to various reasons such as syndromic cases, abnormal karyotype etc.

Database search and generation of control CNV dataset

A set of CNVs present in the general, ostensibly healthy, population with no diagnosis of CHD were derived from five different sources: **a)** 1000 Genome Project Phase 3 dataset, **b)** Database of Genomic variants (DGV), **c)** DECIPHER, **d)** Published literature **e)** Genome Aggregation database (gnomAD), as outlined below. Any CNVs with the same start and end coordinates were treated as the same CNV present in different individuals. CNVs were merged only if they were of the same nature, i.e. either DUP or DEL present in different individuals.

1000 Genome project Phase 3 Controls

The data from the structural analysis group study were available to download on ftp://ftp.1000genomes.ebi.ac.uk/vol1/ftp/phase3/integrated_sv_map/. Multi-allelic CNVs, Alu and L1 insertions, nuclear mitochondrial DNA variants, inversions, and SVA (SINE/VNTR/Alu) composite retrotransposon insertions were excluded from further analysis as this is out of the scope of the current study. Bi-allelic DEL and DUP as well as small DEL from the structural analysis group study were included for further analysis[12]. Additionally, unique CNVs in the marker paper not present in the 1000 Genome Phase 3 structural analysis group study were also included in the control CNV dataset. Sudmant *et al*. study has also identified a list of genes, which were homozygous deleted and thought to be non-essential genes[12]. This list was used to eliminate any of our candidate genes also present in this homozygous deleted gene list.

Database of Genomic variants (DGV) Controls

Control CNV coordinates (GRCh37/hg19 assembly) were downloaded directly from the DGV database (http://dgv.tcag.ca/dgv/app/downloads)[13]. Any CNVs discovered by any or a combination of the following methods were excluded from further analysis due to low breakpoint resolution: bacterial artificial chromosome array comparative genomic hybridization (BAC aCGH), fluorescent *in situ* hybridisation (FISH), polymerase chain reaction (PCR), and representational oligonucleotide microarray analysis (ROMA). Additionally, any CNVs from the 1000 Genome project pilot study and the 1000 Genome project Phase 1 were also excluded from further analysis. We thus included in our analysis CNVs of >6,430 individuals with the exact number of individuals not plausible to determine due to missing ids to a number of CNVs reported in DGV.

DECIPHER controls

We downloaded the DECIPHER population CNV list is available at https://decipher.sanger.ac.uk/about#downloads/data. This list contains population CNVs and their frequencies from the WTCCC2 study (https://www.wtccc.org.uk/ccc2/) and the Deciphering Developmental Disorders (DDD) study. CNVs from the WTCCC2 study were derived from 3,000 samples of the 1958 British Birth Cohort plus 3,000 samples of the UK Blood Service Control group whereas CNVs from the DDD study were derived from the national blood service and ‘’Generation Scotland’’ controls[14, 15].

Controls from published studies

Control CNVs were also available from two out of the nine publications, which were used in this study to update the CHD case CNV dataset. Sanchez-Castro *et al.* is a family trio-based study for which the CNV calls of the unaffected parents were added in the control CNV dataset[10]. Hightower *et al.* study included the CNV calls of non-cardiac patients (other phenotypes like developmental delay), which were also added in our control CNV dataset[9].

gnomAD structural variants

Structural variants of ~11,000 individuals were downloaded from https://gnomad.broadinstitute.org/downloads and DEL and DUP CNVs were used to filtered our final list of candidate genes[16].

CNV identification - Bedtools

All coordinates were converted from previous genome builds to the GRCh37/hg19 genome build using the LiftOver program (https://genome.ucsc.edu/cgi-bin/hgLiftOver). All different genomic analysis using Bedtools as described below, were performed separately for DEL and DUP CNVs, i.e. case DEL CNVs were compared with control DEL CNVs and case DUP CNVs were compared with control DUP CNVs[17]. There is a possibility that DEL and DUP CNVs have different mechanisms of action in the pathogenesis of the disease and they were therefore analysed separately. Using the command Subtract from Bedtools, we identified CNV regions only seen in CHD cases (Supplementary figure 2).

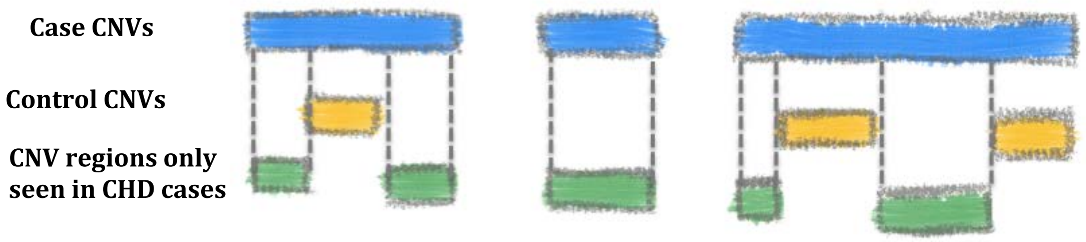

Supplementary figure 2| CNV subtract tool on Bedtools: The blue rectangles represent case CNVs and the yellow ones are control CNVs. The green rectangles are the CNV regions only seen in the CHD cases identified by performing the "subtract" command from BedTools.

Exome data – 829 Tetralogy of Fallot patients

829 cases of sporadic, non-syndromic TOF, of Northern and Western European ancestry, collected in the UK, The Netherlands, Belgium, and Australia as previously described[18], underwent WES at the McGill University/Genome Quebec Innovation Centre, Canada. Exome sequencing was processed using a bioinformatics pipeline using GATK (https://software.broadinstitute.org/gatk/) to call the variants and SnpEff (http://snpeff.sourceforge.net/) to annotate functional consequences.

Filtering of genes

Genes lying within the CNV regions only seen in CHD cases were determined from Ensembl v75 GRCh37/hg19 assembly. The Online Mendelian Inheritance in Man (OMIM) database (downloaded 12/07/2018) was used to filter, retaining only those genes with no previously associated phenotype. Comparison of these genes was

performed against an in-house list of candidate genes (containing novel or rare variants, ExAC frequency <0.01) from the TOF cases. Any genes not present in the TOF exome data with either high (nonsense variants, frameshift, splice variants) or medium (missense, splicing variants) impact variants were excluded from further analysis. Genes in CNV regions only seen in CHD cases were separated into 3 lists: **1)** genes only in DEL CNVs, **2)** genes only in DUP CNVs and **3)** genes in BOTH (included in both DEL and DUP CNVs). Genes from the DUP and BOTH CNVs were included for further analysis if they had high or medium impact SNVs in the WES data. Genes only in DEL and genes in BOTH were only included for further analysis if they had loss-of-function variants in the TOF exome data. Additionally, the following datasets were used to further refine candidate genes: **1)** Exome Aggregation consortium (ExAC browser), **2)** Huang *et al.* – Probability of Haploinsufficiency[19], **3)** RNA-seq data from Zaidi *et al.*[20], **4)** Sudmant *et al.* (Structural analysis group of 1000 Genome Phase 3 project)- list of gene homozygous deleted (nonessential genes)[12], **5)** not present in the list of genes curated from the DDD study, **6)** Ohnologs[21, 22].

CNV counts and intolerance scores were downloaded from (ftp://ftp.broadinstitute.org/pub/ExAC_release/release0.3.1/cnv)[23], which indicate genes that are rarely seen in CNVs in the general population. Some genes do not have an ExAC CNV intolerance score and the reasons are described in Ruderfer *et al.* study[23]. DEL Z-scores were used in genes only in DEL and genes in BOTH CNV regions whereas DUP Z-scores were used in genes in DUP and genes in BOTH CNV regions to further refine candidate genes. The ExAC CNV Z-score thresholds were set by using the top 5% of Z-score values in each of the 3 gene lists. The top 5% ExAC DEL CNV intolerance scores (Z-scores) thresholds were 1.29 and 1.25 for the genes

in DEL and BOTH CNVs, respectively. This was 1.2542 for genes in DUP CNVs and 1.2474 for genes in BOTH CNVs.

Haploinsufficiency scores (version3), as calculated by Huang *et al.*[19], were downloaded from https://decipher.sanger.ac.uk/about#downloads/data. Approximately 10% of all protein-coding genes with pHI value had pHI ≥ 0.65 which was set as the threshold to further refine our candidate gene lists. We have also used the probability of loss-of-function intolerance (pLI) from the ExAC database, where pLI ≥ 0.9 means the gene is less likely to tolerate loss-of-function mutations[24]. However, there were some genes with no ExAC DEL CNV intolerance scores and these were included for further analysis only if they fulfilled the criteria for both pHI and pLI.

A list of genes with their expression values from RNA-seq data in embryonic mice at E9.5 and E14.5 was obtained from Zaidi *et al.* study[20]. The top 25% highly expressed genes in mouse at E9.5 (mean TPM ≥ 2.74) and E14.5 (mean TPM ≥ 3.44) were used as thresholds to further refine our gene lists.

Developmental Disorders Genotype–to-phenotype Database (DDG2P) is a curated list of genes from the Deciphering Developmental Disorders study, which has performed trio-exome sequencing and aCGH of more than 1,000 children in the United Kingdom and Ireland with severe undiagnosed developmental disorders[25, 26]. Any of our final candidate genes present in the DDG2P list of genes were excluded from further analysis. This list was accessed through: https://decipher.sanger.ac.uk/ddd#ddgenes.

Genomics England PanelApp CHD genes

This is a panel of genes for non-syndromic CHD overseen by the NHS Genomic Medicine service that was originally developed for the 100,000 Genomes project. This gene panel consists of green, amber and red list of genes based on high, medium and low evidence, respectively, for association with CHD. In this study, we have used the whole panel as we are using the genes for research purposes. The list of genes can be found at: https://panelapp.genomicsengland.co.uk/panels/212/

Ohnologs

A large scale paralog dataset (Supplementary data D1) containing Ensembl gene name[27] and Paralog status[22, 28] was adapted from Martin-Geary *et al.*[22] using Perl 5, version 18, subversion 2 (v5.18.2)[29]. Paralog status data was obtained by cross-referencing small-scale duplication (SSD) data generated using the FFS method , with whole genome duplication (ohnolog) predictions for human gene pairs obtained from Singh *et al.* [28]. Genes identified by the FFS algorithm as having a partner with exactly one duplication event separating them, which were not present in the ohnolog data, were identified as small-scale duplicates (SSDs). Genes that were present within the ohnolog data were classified as 'strict', intermediate' or 'relaxed' ohnologs depending on the classifiers provided at source. Genes that were not present in either of these two categories were classified as singletons. Duplication partners for SSDs, strict, intermediate, and relaxed ohnologs, were also recorded for each of the Ensembl genes with partners. This data (Supplementary data D1) was subsequently cross referenced with, two test sets containing the CHD genes of interest; 'Case' and 'Genomics England CHD genes' (see earlier methods) (Supplementary data D2:3) using a perl

script (SI P1). This facilitated the analysis of the paralog status and relationship data for these subsets of genes (D4:5). Any genes not present in the dataset set when queried against these two test files were recorded as losses (Case: 7.3%, Genomics England CHD genes: 0%) (D6). A small number of genes contained within the pseudoautosomal (PAR) regions of the Y chromosome were considered diploid-duplicates rather than true paralogs, and were recorded as diploid X genes in separate files for each test set (Supplementary data D7).

Pathway and Gene ontology analysis

Reactome Pathway analysis tool was used with the final 54 candidate genes identified through CNV and WES data in non-syndromic CHD cases. The minimum number of genes per pathway was 5. Also, 8 out of 54 genes were not found in Reactome. Similarly. Ingenuity pathway analysis (IPA) was used with at least 5 genes per pathway[30]. The ratios of the entities found/total entities in each database (Reactome and IPA) were calculated. Gene ontology enrichment analysis for biological process was performed using the final 54 candidate genes[31]. Fisher's exact t-test was selected with p-values corrected with false discovery rate (FDR).

Statistics

All statistical analyses were performed using GraphPad Prism version 8 for MacOS. Chi-square test with Yate's correction and the odds ratio at 95% confidence interval was used to calculate the enrichment of ohnologs in CHD genes compared to the human genome. A p-value of <0.05 was considered statistically significant.

References:

1. Thorsson T, Russell WW, El-Kashlan N, Soemedi R, Levine J, Geisler SB, Ackley T, Tomita-Mitchell A, Rosenfeld JA, Töpf A, et al. Chromosomal Imbalances in Patients with Congenital Cardiac Defects: A Meta-analysis Reveals Novel Potential Critical Regions Involved in Heart Development. *Congenit Heart Dis*. 2015;10:193-208.

2. Firth HV, Richards SM, Bevan AP, Clayton S, Corpas M, Rajan D, Van Vooren S, Moreau Y, Pettett RM and Carter NP. DECIPHER: Database of Chromosomal Imbalance and Phenotype in Humans Using Ensembl Resources. *Am J Hum Genet*. 2009;84:524-33.

3. Kaminsky EB, Kaul V, Paschall J, Church DM, Bunke B, Kunig D, Moreno-De-Luca D, Moreno-De-Luca A, Mulle JG, Warren ST, et al. An evidence-based approach to establish the functional and clinical significance of copy number variants in intellectual and developmental disabilities. *Genet Med*. 2011;13:777-84.

4. Miller DT, Adam MP, Aradhya S, Biesecker LG, Brothman AR, Carter NP, Church DM, Crolla JA, Eichler EE, Epstein CJ, et al. Consensus statement: chromosomal microarray is a first-tier clinical diagnostic test for individuals with developmental disabilities or congenital anomalies. *Am J Hum Genet*. 2010;86:749-64.

5. Vulto-van Silfhout AT, van Ravenswaaij CM, Hehir-Kwa JY, Verwiel ET, Dirks R, van Vooren S, Schinzel A, de Vries BB and de Leeuw N. An update on ECARUCA, the European Cytogeneticists Association Register of Unbalanced Chromosome Aberrations. *Eur J Med Genet*. 2013;56:471-4.

6. Fakhro KA, Choi M, Ware SM, Belmont JW, Towbin JA, Lifton RP, Khokha MK and Brueckner M. Rare copy number variations in congenital heart disease patients identify unique genes in left-right patterning. *Proc Natl Acad Sci U S A*. 2011;108:2915-20.

7. Hitz MP, Lemieux-Perreault LP, Marshall C, Feroz-Zada Y, Davies R, Yang SW, Lionel AC, D'Amours G, Lemyre E, Cullum R, et al. Rare copy number variants contribute to congenital left-sided heart disease. *PLoS Genet*. 2012;8:e1002903.

8. Glessner JT, Bick AG, Ito K, Homsy JG, Rodriguez-Murillo L, Fromer M, Mazaika E, Vardarajan B, Italia M, Leipzig J, et al. Increased frequency of de novo copy number variants in congenital heart disease by integrative analysis of single nucleotide polymorphism array and exome sequence data. *Circ Res*. 2014;115:884-96.

9. Hightower HB, Robin NH, Mikhail FM and Ambalavanan N. Array comparative genomic hybridisation testing in CHD. *Cardiol Young*. 2015;25:1155-72.

10. Sanchez-Castro M, Eldjouzi H, Charpentier E, Busson PF, Hauet Q, Lindenbaum P, Delasalle-Guyomarch B, Baudry A, Pichon O, Pascal C, et al. Search for Rare Copy-Number Variants in Congenital Heart Defects Identifies Novel Candidate Genes and a Potential Role for FOXC1 in Patients With Coarctation of the Aorta. *Circ Cardiovasc Genet*. 2016;9:86-94.

11. Rigler SL, Kay DM, Sicko RJ, Fan R, Liu A, Caggana M, Browne ML, Druschel CM, Romitti PA, Brody LC, et al. Novel copy-number variants in a population-based investigation of classic heterotaxy. *Genet Med*. 2015;17:348-57.

12. Sudmant PH, Rausch T, Gardner EJ, Handsaker RE, Abyzov A, Huddleston J, Zhang Y, Ye K, Jun G, Hsi-Yang Fritz M, et al. An integrated map of structural variation in 2,504 human genomes. *Nature*. 2015;526:75-81.

13. MacDonald JR, Ziman R, Yuen RK, Feuk L and Scherer SW. The Database of Genomic Variants: a curated collection of structural variation in the human genome. *Nucleic Acids Res*. 2014;42:D986-92.

14. Craddock N, Hurles ME, Cardin N, Pearson RD, Plagnol V, Robson S, Vukcevic D, Barnes C, Conrad DF, Giannoulatou E, et al. Genome-wide association study of CNVs in 16,000 cases of eight common diseases and 3,000 shared controls. *Nature*. 2010;464:713-20.

15. Smith BH, Campbell A, Linksted P, Fitzpatrick B, Jackson C, Kerr SM, Deary IJ, Macintyre DJ, Campbell H, McGilchrist M, et al. Cohort Profile: Generation Scotland: Scottish Family Health Study (GS:SFHS). The study, its participants and their potential for genetic research on health and illness. *Int J Epidemiol*. 2013;42:689-700.

16. Collins RL, Brand H, Karczewski KJ, Zhao X, Alföldi J, Khera AV, Francioli LC, Gauthier LD, Wang H, Watts NA, et al. An open resource of structural variation for medical and population genetics. *bioRxiv*. 2019:578674.

17. Quinlan AR. BEDTools: The Swiss-Army Tool for Genome Feature Analysis. *Curr Protoc Bioinformatics*. 2014;47:11.12.1-34.

18. Cordell HJ, Töpf A, Mamasoula C, Postma AV, Bentham J, Zelenika D, Heath S, Blue G, Cosgrove C, Granados Riveron J, et al. Genome-wide association study identifies loci on 12q24 and 13q32 associated with tetralogy of Fallot. *Hum Mol Genet*. 2013;22:1473-81.

19. Huang N, Lee I, Marcotte EM and Hurles ME. Characterising and predicting haploinsufficiency in the human genome. *PLoS Genet*. 2010;6:e1001154.

20. Zaidi S, Choi M, Wakimoto H, Ma L, Jiang J, Overton JD, Romano-Adesman A, Bjornson RD, Breitbart RE, Brown KK, et al. De novo mutations in histone-modifying genes in congenital heart disease. *Nature*. 2013;498:220-3.

21. Singh PP, Affeldt S, Cascone I, Selimoglu R, Camonis J and Isambert H. On the expansion of "dangerous" gene repertoires by whole-genome duplications in early vertebrates. *Cell Rep*. 2012;2:1387-98.

22. Martin-Geary A, Reardon M, Keith B, Tassabehji M and Robertson DL. Human genetic disease is greatly influenced by the underlying fragility of evolutionarily ancient genes. *bioRxiv*. 2019:558916.

23. Ruderfer DM, Hamamsy T, Lek M, Karczewski KJ, Kavanagh D, Samocha KE, Daly MJ, MacArthur DG, Fromer M, Purcell SM, et al. Patterns of genic intolerance of rare copy number variation in 59,898 human exomes. *Nat Genet*. 2016;48:1107-11.

24. Lek M, Karczewski KJ, Minikel EV, Samocha KE, Banks E, Fennell T, O'Donnell-Luria AH, Ware JS, Hill AJ, Cummings BB, et al. Analysis of protein-coding genetic variation in 60,706 humans. *Nature*. 2016;536:285-91.

25. Wright CF, Fitzgerald TW, Jones WD, Clayton S, McRae JF, van Kogelenberg M, King DA, Ambridge K, Barrett DM, Bayzetinova T, et al. Genetic diagnosis of developmental disorders in the DDD study: a scalable analysis of genome-wide research data. *Lancet*. 2015;385:1305-14.

26. Study DDD. Large-scale discovery of novel genetic causes of developmental disorders. *Nature*. 2015;519:223-8.

27. Herrero J, Muffato M, Beal K, Fitzgerald S, Gordon L, Pignatelli M, Vilella AJ, Searle SM, Amode R, Brent S, et al. Ensembl comparative genomics resources. *Database (Oxford)*. 2016;2016.

28. Singh PP, Arora J and Isambert H. Identification of Ohnolog Genes Originating from Whole Genome Duplication in Early Vertebrates, Based on Synteny Comparison across Multiple Genomes. *PLoS Comput Biol*. 2015;11:e1004394.

29. Wall L. *Programming Perl*: O'Reilly & Associates, Inc., Sebastopol, CA, USA; 2000.

30. Krämer A, Green J, Pollard J and Tugendreich S. Causal analysis approaches in Ingenuity Pathway Analysis. *Bioinformatics*. 2014;30:523-30.

31. Mi H, Huang X, Muruganujan A, Tang H, Mills C, Kang D and Thomas PD. PANTHER version 11: expanded annotation data from Gene Ontology and Reactome pathways, and data analysis tool enhancements. *Nucleic Acids Res*. 2017;45:D183-D189.
