# Supplementary material for: Integration of Large-Scale Genomic Data Sources With Evolutionary History Reveals Novel Genetic Loci for Congenital Heart Disease
Source: Circ Genom Precis Med. 2019 Oct 15;12(10):e002694. doi: 10.1161/CIRCGEN.119.002694 (PMC6798745; doi:10.1161/CIRCGEN.119.002694)
Supplement: Supplementary file 4 [file hcg-12-e002694-s004.pdf]

Integration of Large-scale Genomic Data Sources with Evolutionary History Reveals Novel Genetic Loci for Congenital Heart Disease

Running title: *Fotiou et al.; Ohnologs in congenital heart disease*

Elisavet Fotiou, MRes[1], Simon Williams, PhD[1], Alexandra Martin-Geary, MSc[2], David L. Robertson, PhD[2,3], Gennadiy Tenin, PhD[1], Kathryn E. Hentges, PhD[2], Bernard Keavney, MD[1,4]

[1]Division of Cardiovascular Sciences, School of Medical Sciences, Faculty of Biology, Medicine, and Health, Manchester Academic Health Science Centre, University of Manchester, Manchester, UK, [2]Division of Evolution and Genomic science, University of Manchester, Manchester, [3]MRC-University of Glasgow Centre for Virus Research, Glasgow, [4]Manchester Heart Centre, Manchester University NHS Foundation Trust, Manchester

American Heart Association.
Circulation: Genomic and Precision Medicine

Correspondence:

Professor Bernard D. Keavney
The University of Manchester
AV Hill Building
Manchester, M13 9PT
United Kingdom
Tel: 44 (0)161 275 1201
Email: bernard.keavney@manchester.ac.uk

Miss Elisavet Fotiou
The University of Manchester
AV Hill Building
Manchester, M13 9PT
United Kingdom
Tel: 01612750238
Elisavet.fotiou@postgrad.manchester.ac.uk

Journal Subject Terms: Genetics; Congenital Heart Disease

Abstract:

Background - Most cases of congenital heart disease (CHD) are sporadic and non-syndromic, with poorly understood aetiology. Rare genetic variants have been found to affect the risk of sporadic, non-syndromic CHD, but individual studies to date are of only moderate sizes, and none to date has incorporated the ohnolog status of candidate genes in the analysis. Ohnologs are genes retained from ancestral whole-genome duplications during evolution; multiple lines of evidence suggest ohnologs are over-represented among dosage-sensitive genes. We integrated large-scale data on rare variants with evolutionary information on ohnolog status to identify novel genetic loci predisposing to CHD.

Methods - We compared copy number variants (CNVs) present in 4,634 non-syndromic CHD cases derived from publicly available data resources and the literature, and >27,000 healthy individuals. We analysed deletions and duplications independently and identified CNV regions exclusive to cases. These data were integrated with whole-exome sequencing data from 829 sporadic, non-syndromic patients with Tetralogy of Fallot. We placed our findings in an evolutionary context by comparing the proportion of vertebrate ohnologs in CHD cases and controls.

Results - Novel genetic loci in CHD cases were significantly enriched for ohnologs compared to the genome (χ^2-test, $p<0.0001$, OR=1.253, 95% CI:1.199-1.309). We identified 54 novel candidate protein-coding genes supported by both: (i) CNV and whole-exome sequencing data; and (ii) ohnolog status.

Conclusions - We have identified new CHD candidate loci, and show for the first time that ohnologs are over-represented among CHD genes. Incorporation of evolutionary metrics may be useful in refining candidate genes emerging from large-scale genetic evaluations of CHD.

American Heart Association
Circulation: Genomic and Precision Medicine

Key words: congenital heart disease; copy number variant; single nucleotide polymorphism; ohnologs, whole-exome sequencing

Non-standard Abbreviations and Acronyms

CHD	Congenital heart disease
CNV	Copy number variant
DEL	Deletion
DUP	Duplication
SNV	Single nucleotide variant
SSD	Small-scale duplication
TOF	Tetralogy of Fallot
WES	Whole exome sequencing
WGD	Whole-genome duplication

American Heart Association
Circulation: Genomic and Precision Medicine

Background

Congenital heart disease (CHD) is the most prevalent birth defect in humans, occurring in approximately 8 per 1000 live births, and consisting of malformation of the heart and/or the great vessels[1]. Around 20% of all CHDs can be attributed to chromosomal imbalances such as Down and Turner, and 22q11 deletion syndromes; around 80% occur as sporadic, non-syndromic CHD. In such cases, CHD behaves overall as a genetically complex trait with moderate heritability. Previous genome-wide investigations into CHD have found evidence for rare causative copy number variants (CNVs) and single nucleotide variants (SNVs); and associations with common SNVs in GWAS[2-6]. It has been estimated in previous studies that several hundred genes may be involved in polygenic CHD susceptibility; therefore, many remain to be discovered[7].

CNVs are 1 kilobase (kb) to several megabase (Mb) sized regions of duplication (DUP) and deletion (DEL) in the genome. A 2014 meta-analysis of CNVs in 1694 non-syndromic CHD cases identified 79 chromosomal regions in which 5 or more CHD cases had overlapping imbalances[5]. The estimated prevalence of pathogenic CNVs in non-syndromic CHD patients is 4-14%, whereas in syndromic CHD patients it is 15-20% (the most common being 22q11 deletion syndrome)[3, 8, 9]. There are multiple mechanisms by which a CNV may lead to disease including the disruption of chromosome structure, alteration of gene expression due to disruption of regulatory elements, and changes of the relative amounts of dosage-sensitive genes[10].

American Heart Association
Circulation: Genomic and Precision Medicine

The dosage-balance model postulates that, for genes that are in stoichiometric relationships (for example forming protein complexes with other genes), any perturbation in their relative ratios will tend to be deleterious[10]. In the early course of vertebrate evolution, around 500 million years ago, two whole-genome duplications (WGD), during which gene stoichiometry throughout the genome was preserved, as all genes were duplicated, took place.

Periods of gene loss followed each of these events, resulting in the retention of some WGD paralogs in the genome (termed "ohnologs") and the loss of others. The dosage-balance model would predict that ohnologs should be enriched for dosage-sensitive genes. [11] Ohnologs, of which there are around 7,000 in the human genome, have indeed been shown to exhibit characteristics consistent with dosage-sensitivity: for example, ohnologs are enriched for haploinsufficient genes[11,12]; and Makino *et al*. reported, based on CNV data in healthy individuals from the Database of Genomic Variants (DGV), that genomic regions (~2Mb in size) near ohnologs are CNV deserts, indicating that those regions are dosage-balanced[13].

The formation and fixation of gene duplications within the genome is subject to different evolutionary mechanisms –small scale duplications (SSD) involving relatively few genes, and WGD. A strong relationship between the evolutionary mechanism of duplication and phenotypic consequences, including heritable diseases, has been previously shown[14 15, 16]. Ohnologs have a significant association with certain human genetic diseases; for example 12 out of 16 reported candidate genes within the Down syndrome critical region (21q22.12, 21q22.13 and 21q22.2) are dosage-balanced ohnologs[11]. By contrast, genes arising from SSDs lack enrichment for disease association [17]. In addition, ohnologs are enriched for genes involved in signalling and gene regulation, key cardiovascular developmental processes[11]. These considerations led us to hypothesise that ohnologs may be enriched among CHD causative genes.

American Heart Association
Circulation: Genomic and Precision Medicine

We tested this hypothesis in a meta-analysis of CNV data including 4,634 non-syndromic CHD cases, and integrated these data with a whole-exome sequencing (WES) study of 829 cases of Tetralogy of Fallot (TOF), the commonest cyanotic CHD phenotype, which has been previously shown to have a significant aetiological contribution from CNVs[6]. These were compared with control data, which were derived from large-scale genomic resources[18-21].

Methods

The appropriate institutional review bodies approved all recruitment of human participants in this study. The study corresponded with the stipulations of the Declaration of Helsinki, and all participants (or their parents, if affected probands were children too young to themselves consent) provided informed consent. Data from consortia were accessed subject to the applicable data-sharing agreements. Summary data, analytic methods, and summary study materials will be made available to other researchers for purposes of reproducing the results or replicating the analyses reported here, on request to the corresponding authors. Full Materials and Methods are available in the Data supplement of the article.

American Heart Association®
Circulation: Genomic and Precision Medicine

Results

Update of CHD CNV dataset and generation of control CNV dataset

We updated the previous meta-analysis of CNVs in non-syndromic CHD cases[5], in a further 2,882 non-syndromic CHD cases from DECIPHER, ECARUCA and ISCA databases and further published studies investigating the role of CNVs in CHD[4, 20, 22-32]. The updated CHD case CNV dataset consists of 4,634 unrelated individuals of different ancestries (Table 1). The outline workflow to identify candidate genes is shown in Figure 1. Filtering of the CHD case population against DECIPHER known microdeletion/microduplication syndromes resulted in 224 cases being removed; this left 4,410 CHD cases with 3,362 DEL CNVs and 2,540 DUP CNVs which were used for further analysis (Supplementary figure 1).

A control CNV dataset was generated by acquiring CNV data from individuals not explicitly identified as having a developmental disorder, who were enrolled in the 1000 Genome Project Phase 3, DGV, DECIPHER, and published studies[21, 27, 28, 33, 34]. The control CNV dataset

resulted in 256,511 DEL CNVs, 84,343 DUP CNVs and 6,403 BOTH CNVs, i.e. either DEL or DUP. gnomAD CNVs[35] were incorporated into the analysis as they became available, and resulted in an additional 51,420 DUP CNVs and 198,611 DEL CNVs.

Comparison of CHD CNV regions with control CNV regions

All CHD DEL and DUP CNV regions (coordinates hg19) were compared against control DEL and DUP CNV regions, respectively. Any CHD CNV regions overlapping control CNV regions were excluded. As a result, we identified DEL and DUP CNV regions only seen in non-syndromic CHD cases. The genes located in those regions were annotated using the Ensembl database. There were a number of genes that already had an assigned phenotype (OMIM)[36]; among these, 59 had been previously associated with CHD pathogenesis such as *ZIC3*, *NKX2-6*, *GATA4*, *JAG1*, *GJA1* and *TBX5*. All genes with OMIM assigned phenotypes were excluded from further analysis.

American Heart Association
Circulation: Genomic and Precision Medicine

Novel genes found in the CNV regions only seen in CHD cases were then compared to an in-house list of 12,771 genes with novel or rare SNVs (either absent from ExAC or with frequency of <0.01) from WES data in 829 TOF cases[6]. Genes supported by both CNV and WES data were included for further analysis. In total, 3,082 genes in DEL CNVs, 4,297 genes in DUP CNVs and 3,068 in BOTH CNVs (i.e. genes found in DEL and DUP CNVs) were also found in the TOF WES data with either high (nonsense variants, frameshift, splice variants) or medium (missense, splice variants) impact SNVs. This intersection of CNV and WES data led to an overall reduction of ~60% in the number of candidate genes for CHD (Figure 2).

Ohnologs are highly enriched in CHD cases whereas small-scale duplications (SSD) and singleton genes are not.

Ohnologs (N=7,023) were identified using data from Singh *et al* (2015)[37], available at http://ohnologs.curie.fr/. SSDs (N=7,014) were extracted from Ensembl gene trees[12]. Any remaining genes that were neither found in the ohnolog dataset nor identified as having a direct paralog were considered for the purpose of this study to be singletons. The frequencies of ohnologs, SSDs and singletons among the candidate CHD genes were compared with their frequency in the human genome. Novel genes supported by the CNV data in CHD cases were found to be enriched for ohnologs (14.65% vs 12.05%, χ^2 test, p<0.0001, OR=1.253, 95% CI: 1.199-1.309,) (Figure 3A). There were no differences in SSDs (Figure 3B) and an under-representation for singletons (Figure 3C) compared to the human genome. There was a 2.3-fold increased enrichment of ohnologs in the genes supported by both CNV and WES data in CHD cases (χ^2 test, p< 0.0001, OR=3.751, 95% CI: 3.574-3.937). In this instance, SSDs were also enriched in CHD cases compared to the human genome (χ^2 test, p< 0.0001, OR=1.437, 95% CI: 1.356-1.905). However, ohnologs were 2-times elevated compared to SSD genes (33.94% versus 16.43%). Additionally, we assessed our methodology by applying it to a group of genes with strong *a priori* evidence for pathogenicity. The crowd-sourced Genomics England "PanelApp" gene list for CHD (available at https://panelapp.genomicsengland.co.uk/panels/212/), which represents a consensus view of causative genes, was highly enriched for ohnologs (76.6% vs 12.05%, χ^2 test, p<0.0001, OR=23.89, 95% CI: 12.33-46.18). We therefore used ohnolog status as an additional candidate gene filter.

American Heart Association
Circulation: Genomic and Precision Medicine

Candidate genes supported by both CNV and WES data of CHD cases

In order to further refine our candidate genes, we integrated additional genomic resources including the top 5% ExAC CNV intolerance scores, probability of haploinsuffieciency (pHI)[38], probability of loss-of-function intolerance (pLI)[19], and RNAseq expression data from mouse embryonic hearts[39]. Lastly, we incorporated ohnolog status. Genes from BOTH CNVs were analysed twice; once with the metrics used for genes from DEL CNVs and once with the metrics used for genes from DUP CNVs (Figure 4).

This led to the identification of 9 candidate genes from DEL and BOTH CNVs: *BRWD1, DIP2C, EYA3, GRB10, HNRNPC, RC3H2, SLIT3, TLN1* and *UBASH3B.* All 9 have the following properties: a) loss-of-function (LoF) variants in the WES data, b) found in DEL or BOTH CNV regions only seen in non-syndromic CHD cases, c) top 5% of ExAC DEL CNV intolerance scores, d) haploinsufficient (pHI≥0.65) and/or unable to tolerate LoF variants (pLI≥0.9), e) in the top 25% of highly expressed genes in mouse heart at E9.5 and/or E14.5, f) ohnolog, g) not present in the list of genes curated from the DDD study, h) not classified as human non-essential genes from the Sudmant study[21] (Table 2).

In addition, we found 45 candidate genes from DUP and BOTH CNVs, which had the following properties: a) high or medium impact SNVs in the WES data, b) found in DUP and BOTH CNV regions only seen in non-syndromic CHD cases, c) top 5% of ExAC DUP CNV intolerance scores, d) in the top 25% of highly expressed genes in mouse heart at E9.5 and/or E14.5, e) ohnolog, f) not present in the list of genes curated from the DDD study, g) not in the list of non-essential human genes from the Sudmant study[21] (Table 2).

American Heart Association.
Circulation: Genomic and Precision Medicine

Pathway enrichment and gene ontology analysis

We performed pathway enrichment analysis, using the Reactome Pathways Analysis tool[40], on the final 54 candidate genes supported by both CNV and WES data in non-syndromic CHD cases. This resulted in 11 pathways, where >5 of our candidate genes were involved in those pathways (Table 3). The top 3 pathways based on entities ratio (entities found/total entities) from Reactome were ‘’axon guidance’’, ‘’signalling by receptor tyrosine kinases’’ and ‘’cellular responses to external stimuli’’. In addition, Ingenuity pathway analysis (IPA) was also used with the only pathway including >5 genes being ‘’axon guidance signalling’’. Gene ontology analysis[41] of our candidate genes revealed 22 Gene ontology (GO) terms with particular enrichment on 4 GO terms; apoptotic process involved in luteolysis (GO0061364) (FDR corrected p-value= 0.0462), ventricular septum morphogenesis (GO0060412) (FDR corrected p-value=0.00921), ventricular septum development (GO0003281) (FDR corrected p-value=0.0343) and cardiac septum morphogenesis (GO0060411) (FDR corrected p-value=0.036). Both pathway and gene ontology analysis identified processes in which the genes *ABLIM1*, *ARHGEF12*, *SLIT2* and *SLIT3* are involved (Figure 5).

American Heart
Circulation: Genomic and Precision Medicine

***SLIT2* and *SLIT3* variants in CHD**

SLIT2 and *SLIT3* were the most strongly supported genes found both by pathway analysis and gene ontology (Figure 5). Therefore, we further explored the phenotypic associations of these genes within our population.

In the present study, individuals with CNVs including *SLIT3* were reported with malformation of the heart and great vessels (n=1), VSD (n=1), atrial septal defect (n=3) and TOF (n=1) whereas individuals with *SLIT2* CNVs were reported with malformation of the heart and great vessels (n=1), VSD (n=2) and double outlet right ventricle (n=1). In addition, 20 missense

SNVs and 3 splice-site SNVs in *SLIT3* were found in 24 out of 829 TOF cases (2.9%, 95% CI: 1.91%-4.35%) and *SLIT2* had 12 missense SNVs and 2 splice-site SNVs in 14 out of 829 TOF cases (1.7%, 95% CI: 0.9%-2.9%). Probands were available for 12 *SLIT3* variants and 5 *SLIT2* variants which were confirmed by Sanger sequencing. Remaining variants were confirmed to have good coverage using Integrative Genomics Viewer (IGV). Samples from both parents were available for 9 probands with *SLIT3* variants and were analysed for variant inheritance; 2 of the 9 *SLIT3* variants tested were identified as *de novo*. Samples from both parents were available for 5 probands with *SLIT2* variants and were all either maternally or paternally inherited.

Discussion

American Heart Association. Circulation: Genomic and Precision Medicine

Here, we performed a large-scale genome-wide meta-analysis study of non-syndromic CHD cases and identified 54 novel candidate genes for CHD. In addition to the large size of our dataset, we incorporated a novel analysis strategy incorporating the evolutionary origin of gene duplications. Ohnologs tend not to be observed in CNVs in vertebrate genomes[13]. Moreover, McLysaght *et al.* have also shown that ohnologs are significantly overrepresented in pathogenic CNVs associated with schizophrenia and neurodevelopmental disorders and that they are the possible cause of the deleterious effects of these rare pathogenic CNVs[14]. Here, we have shown for the first time that genes included in CNVs from CHD cases are significantly enriched for ohnologs compared to the human genome. Due to this significant association between ohnologs and CHD we incorporated ohnolog status in our methodology to identify novel genetic loci associated with CHD.

Pathway analysis and gene ontology analysis concordantly identified the *SLIT2* and *SLIT3* genes, which have recently received increasing attention in heart development[42]. In

vertebrates, the Slit family comprises of 3 known members (SLIT1-3), which are highly conserved secreted proteins that bind to Roundabout (ROBO) receptors. *SLIT2* and *SLIT3* are expressed during mouse embryonic development and interact with ROBO1 and ROBO2[43, 44]. They both encode proteins that consist of 4 LRR domains (leucine-rich repeats) also called D1-D4, 8 EGF repeats (epidermal growth factor) and 1 Laminin-G-like domain[43]. *Slit3* is expressed early in murine cardiogenesis in the cardiac crescent at E7.5 and linear heart tube at E8.5, later expression being restricted to the myocardium of the atria and OFT but not in the cardiac cushions or valves[44, 45]. *Slit2* is strongly expressed in the pharyngeal region at E8.5-E9.5 and later in the ventricular trabecular myocardium, epicardium, aortic semilunar valves and the mesenchyme surrounding the caval veins[44, 46]. *Slit3*$^{-/-}$ mutant mice exhibit ventricular septal defect (VSD), thick atrioventricular valves and hypoplastic posterior aortic semilunar leaflet with Slit2$^{-/-}$ mutant mice exhibiting bicuspid aortic valves and immature semilunar valves[44]. Robo1$^{-/-}$ mutant mice also exhibit VSD with down-regulation of NOTCH signalling, suggesting a potential mechanism for the underlying defects[44]. In another study, *Slit3*$^{-/-}$ mice also exhibit congenital diaphragmatic hernia[47]. Congenital heart defects ranging from bicuspid aortic valves to septal and outflow tract defects are therefore observed in variety of animal models in which genes involved in the SLIT/ROBO pathway have been inactivated.

We identified *SLIT2* and *SLIT3* heterozygous SNVs in 2.9% and 1.7% non-syndromic TOF patients, respectively. All SNVs were novel or rare (either absent from ExAC or with frequency of <0.01) and predicted with *in silico* tools to be pathogenic. The majority of the SNVs in both genes were missense, although a few splice site SNVs were also found. Their functional relevance will be of interest in future studies. Both genes were also present in CNVs in CHD patients with varying phenotypes including septal defects and malformation of the great

American Heart Association
Circulation: Genomic and Precision Medicine

arteries. This is the first study to find an association of *SLIT2* and *SLIT3* with predisposition to human CHD, although, of note, a recent study identified *ROBO1* LOF SNVs in cases with TOF and septal defects[48].

Limitations

This study has certain limitations. The databases and publications included in this analysis incorporated different CNV detection platforms and analysis algorithms[49]. Irrespective of the method used in the studies identifying pathogenic CNVs, we only included studies that used the same genotyping method between cases and controls and confirmed their CNV detection by an additional methodology like qPCR, which to a degree addresses this limitation. Another potential limitation is the fact that during our filtering strategy we might have missed some important genes crucial for cardiac development. Though we accept that all important genes will not have been captured, we detected 54 strong candidate genes supported by multiple lines of evidence as having a causative role in non-syndromic CHD. Further research in much larger numbers of comprehensively genetically characterised CHD cases is warranted to establish the magnitude of the contribution of these genes, and to discover novel loci.

Circulation: Genomic and Precision Medicine

In conclusion, we show that ohnologs are over-represented in CHD cases and that incorporation of the evolutionary origins of genes is useful in refining candidate genes emerging from large-scale genetic evaluations of CHD. We also observe that CNVs and SNVs in *SLIT2* and *SLIT3* are associated with CHD involving TOF, septal defects and outflow tract defects, supporting the importance of the SLIT-ROBO signalling pathway in heart development.

Acknowledgments: This study makes use of data generated by the DECIPHER Consortium. A full list of centres that contributed to the generation of the data is available from

https://decipher.sanger.ac.uk/ and via email from decipher@sanger.ac.uk. Funding for the DECIPHER project was provided by the Wellcome Trust[20].

Sources of Funding: Supported by the British Heart Foundation. AM-G is funded by a PhD studentship from the Medical Research Council (#1622139). DLR is partially funded by the Medical Research Council (MC UU 1201412). BK holds a British Heart Foundation Personal Chair

Disclosures: None.

References:

1. Liu Y, Chen S, Zühlke L, Black GC, Choy MK, Li N, Keavney BD. Global birth prevalence of congenital heart defects 1970-2017: updated systematic review and meta-analysis of 260 studies. *Int J Epidemiol*. 2019.

2. Cordell HJ, Bentham J, Topf A, Zelenika D, Heath S, Mamasoula C, Cosgrove C, Blue G, Granados-Riveron J, Setchfield K, et al. Genome-wide association study of multiple congenital heart disease phenotypes identifies a susceptibility locus for atrial septal defect at chromosome 4p16. *Nat Genet*. 2013;45:822-4.

3. Soemedi R, Wilson IJ, Bentham J, Darlay R, Töpf A, Zelenika D, Cosgrove C, Setchfield K, Thornborough C, Granados-Riveron J, et al. Contribution of global rare copy-number variants to the risk of sporadic congenital heart disease. *Am J Hum Genet*. 2012;91:489-501.

4. Kaminsky EB, Kaul V, Paschall J, Church DM, Bunke B, Kunig D, Moreno-De-Luca D, Moreno-De-Luca A, Mulle JG, Warren ST, et al. An evidence-based approach to establish the functional and clinical significance of copy number variants in intellectual and developmental disabilities. *Genet Med*. 2011;13:777-84.

5. Thorsson T, Russell WW, El-Kashlan N, Soemedi R, Levine J, Geisler SB, Ackley T, Tomita-Mitchell A, Rosenfeld JA, Töpf A, et al. Chromosomal Imbalances in Patients with Congenital Cardiac Defects: A Meta-analysis Reveals Novel Potential Critical Regions Involved in Heart Development. *Congenit Heart Dis*. 2015;10:193-208.

6. Page DJ, Miossec MJ, Williams SG, Monaghan RM, Fotiou E, Cordell H, Sutcliffe L, Topf A, Bourgey M, Bourque G, et al. Whole Exome Sequencing Reveals the Major Genetic Contributors to Non-Syndromic Tetralogy of Fallot. *Circ Res*. 2018.

American Heart Association
Circulation: Genomic and Precision Medicine

7. Jin SC, Homsy J, Zaidi S, Lu Q, Morton S, DePalma SR, Zeng X, Qi H, Chang W, Sierant MC, et al. Contribution of rare inherited and de novo variants in 2,871 congenital heart disease probands. *Nat Genet*. 2017;49:1593-1601.

8. Greenway SC, Pereira AC, Lin JC, DePalma SR, Israel SJ, Mesquita SM, Ergul E, Conta JH, Korn JM, McCarroll SA, et al. De novo copy number variants identify new genes and loci in isolated sporadic tetralogy of Fallot. *Nat Genet*. 2009;41:931-5.

9. Costain G, Roche SL, Scherer SW, Silversides CK, Bassett AS. Rare copy number variations in an adult with transposition of the great arteries emphasize the importance of updated genetic assessments in syndromic congenital cardiac disease. *Int J Cardiol*. 2016;203:516-8.

10. Rice AM, McLysaght A. Dosage sensitivity is a major determinant of human copy number variant pathogenicity. *Nat Commun*. 2017;8:14366.

11. Makino T, McLysaght A. Ohnologs in the human genome are dosage balanced and frequently associated with disease. *Proc Natl Acad Sci U S A*. 2010;107:9270-4.

12. Martin-Geary A, Reardon M, Keith B, Tassabehji M, Robertson DL. Human genetic disease is greatly influenced by the underlying fragility of evolutionarily ancient genes. *bioRxiv*. 2019:558916.

13. Makino T, McLysaght A, Kawata M. Genome-wide deserts for copy number variation in vertebrates. *Nat Commun*. 2013;4:2283.

14. McLysaght A, Makino T, Grayton HM, Tropeano M, Mitchell KJ, Vassos E, Collier DA. Ohnologs are overrepresented in pathogenic copy number mutations. *Proc Natl Acad Sci U S A*. 2014;111:361-6.

15. Smith BH, Campbell A, Linksted P, Fitzpatrick B, Jackson C, Kerr SM, Deary IJ, Macintyre DJ, Campbell H, McGilchrist M, et al. Cohort Profile: Generation Scotland: Scottish Family Health Study (GS:SFHS). The study, its participants and their potential for genetic research on health and illness. *Int J Epidemiol*. 2013;42:689-700.

16. Dickerson JE, Robertson DL. On the origins of Mendelian disease genes in man: the impact of gene duplication. *Mol Biol Evol*. 2012;29:61-9.

17. Singh PP, Affeldt S, Malaguti G, Isambert H. Human dominant disease genes are enriched in paralogs originating from whole genome duplication. *PLoS Comput Biol*. 2014;10:e1003754.

18. Ruderfer DM, Hamamsy T, Lek M, Karczewski KJ, Kavanagh D, Samocha KE, Daly MJ, MacArthur DG, Fromer M, Purcell SM, et al. Patterns of genic intolerance of rare copy number variation in 59,898 human exomes. *Nat Genet*. 2016;48:1107-11.

American Heart Association
Circulation: Genomic and Precision Medicine

19. Lek M, Karczewski KJ, Minikel EV, Samocha KE, Banks E, Fennell T, O'Donnell-Luria AH, Ware JS, Hill AJ, Cummings BB, et al. Analysis of protein-coding genetic variation in 60,706 humans. *Nature*. 2016;536:285-91.

20. Bragin E, Chatzimichali EA, Wright CF, Hurles ME, Firth HV, Bevan AP, Swaminathan GJ. DECIPHER: database for the interpretation of phenotype-linked plausibly pathogenic sequence and copy-number variation. *Nucleic Acids Res*. 2014;42:D993-D1000.

21. Sudmant PH, Rausch T, Gardner EJ, Handsaker RE, Abyzov A, Huddleston J, Zhang Y, Ye K, Jun G, Hsi-Yang Fritz M, et al. An integrated map of structural variation in 2,504 human genomes. *Nature*. 2015;526:75-81.

22. Vulto-van Silfhout AT, van Ravenswaaij CM, Hehir-Kwa JY, Verwiel ET, Dirks R, van Vooren S, Schinzel A, de Vries BB, de Leeuw N. An update on ECARUCA, the European Cytogeneticists Association Register of Unbalanced Chromosome Aberrations. *Eur J Med Genet*. 2013;56:471-4.

23. Fakhro KA, Choi M, Ware SM, Belmont JW, Towbin JA, Lifton RP, Khokha MK, Brueckner M. Rare copy number variations in congenital heart disease patients identify unique genes in left-right patterning. *Proc Natl Acad Sci U S A*. 2011;108:2915-20.

24. Hitz MP, Lemieux-Perreault LP, Marshall C, Feroz-Zada Y, Davies R, Yang SW, Lionel AC, D'Amours G, Lemyre E, Cullum R, et al. Rare copy number variants contribute to congenital left-sided heart disease. *PLoS Genet*. 2012;8:e1002903.

25. Glessner JT, Bick AG, Ito K, Homsy JG, Rodriguez-Murillo L, Fromer M, Mazaika E, Vardarajan B, Italia M, Leipzig J, et al. Increased frequency of de novo copy number variants in congenital heart disease by integrative analysis of single nucleotide polymorphism array and exome sequence data. *Circ Res*. 2014;115:884-96.

26. Rigler SL, Kay DM, Sicko RJ, Fan R, Liu A, Caggana M, Browne ML, Druschel CM, Romitti PA, Brody LC, et al. Novel copy-number variants in a population-based investigation of classic heterotaxy. *Genet Med*. 2015;17:348-57.

27. Hightower HB, Robin NH, Mikhail FM, Ambalavanan N. Array comparative genomic hybridisation testing in CHD. *Cardiol Young*. 2015;25:1155-72.

28. Sanchez-Castro M, Eldjouzi H, Charpentier E, Busson PF, Hauet Q, Lindenbaum P, Delasalle-Guyomarch B, Baudry A, Pichon O, Pascal C, et al. Search for Rare Copy-Number Variants in Congenital Heart Defects Identifies Novel Candidate Genes and a Potential Role for FOXC1 in Patients With Coarctation of the Aorta. *Circ Cardiovasc Genet*. 2016;9:86-94.

29. Hanchard NA, Umana LA, D'Alessandro L, Azamian M, Poopola M, Morris SA, Fernbach S, Lalani SR, Towbin JA, Zender GA, et al. Assessment of large copy number variants in patients with apparently isolated congenital left-sided cardiac lesions reveals clinically relevant genomic events. *Am J Med Genet A*. 2017;173:2176-2188.

Circulation: Genomic and Precision Medicine

30. Xie L, Chen JL, Zhang WZ, Wang SZ, Zhao TL, Huang C, Wang J, Yang JF, Yang YF, Tan ZP. Rare de novo copy number variants in patients with congenital pulmonary atresia. *PLoS One*. 2014;9:e96471.

31. Xie HM, Werner P, Stambolian D, Bailey-Wilson JE, Hakonarson H, White PS, Taylor DM, Goldmuntz E. Rare copy number variants in patients with congenital conotruncal heart defects. *Birth Defects Res*. 2017;109:271-295.

32. Miller DT, Adam MP, Aradhya S, Biesecker LG, Brothman AR, Carter NP, Church DM, Crolla JA, Eichler EE, Epstein CJ, et al. Consensus statement: chromosomal microarray is a first-tier clinical diagnostic test for individuals with developmental disabilities or congenital anomalies. *Am J Hum Genet*. 2010;86:749-64.

33. MacDonald JR, Ziman R, Yuen RK, Feuk L, Scherer SW. The Database of Genomic Variants: a curated collection of structural variation in the human genome. *Nucleic Acids Res*. 2014;42:D986-92.

34. Auton A, Brooks LD, Durbin RM, Garrison EP, Kang HM, Korbel JO, Marchini JL, McCarthy S, McVean GA, Abecasis GR, et al. A global reference for human genetic variation. *Nature*. 2015;526:68-74.

35. Collins RL, Brand H, Karczewski KJ, Zhao X, Alföldi J, Khera AV, Francioli LC, Gauthier LD, Wang H, Watts NA, et al. An open resource of structural variation for medical and population genetics. *bioRxiv*. 2019:578674.

36. McKusick-Nathans Institute of Genetic Medicine JHUB, MD). Online Mendelian Inheritance in Man, OMIM ®. 2019.

37. Singh PP, Arora J, Isambert H. Identification of Ohnolog Genes Originating from Whole Genome Duplication in Early Vertebrates, Based on Synteny Comparison across Multiple Genomes. *PLoS Comput Biol*. 2015;11:e1004394.

38. Huang N, Lee I, Marcotte EM, Hurles ME. Characterising and predicting haploinsufficiency in the human genome. *PLoS Genet*. 2010;6:e1001154.

39. Zaidi S, Choi M, Wakimoto H, Ma L, Jiang J, Overton JD, Romano-Adesman A, Bjornson RD, Breitbart RE, Brown KK, et al. De novo mutations in histone-modifying genes in congenital heart disease. *Nature*. 2013;498:220-3.

40. Fabregat A, Jupe S, Matthews L, Sidiropoulos K, Gillespie M, Garapati P, Haw R, Jassal B, Korninger F, May B, et al. The Reactome Pathway Knowledgebase. *Nucleic Acids Res*. 2018;46:D649-D655.

American Heart Association
Circulation: Genomic and Precision Medicine

41. Mi H, Huang X, Muruganujan A, Tang H, Mills C, Kang D, Thomas PD. PANTHER version 11: expanded annotation data from Gene Ontology and Reactome pathways, and data analysis tool enhancements. *Nucleic Acids Res*. 2017;45:D183-D189.

42. Zhao J, Mommersteeg MTM. Slit-Robo signalling in heart development. *Cardiovasc Res*. 2018;114:794-804.

43. Blockus H and Chédotal A. Slit-Robo signaling. *Development*. 2016;143:3037-44.

44. Mommersteeg MT, Yeh ML, Parnavelas JG, Andrews WD. Disrupted Slit-Robo signalling results in membranous ventricular septum defects and bicuspid aortic valves. *Cardiovasc Res*. 2015;106:55-66.

45. Medioni C, Bertrand N, Mesbah K, Hudry B, Dupays L, Wolstein O, Washkowitz AJ, Papaioannou VE, Mohun TJ, Harvey RP, et al. Expression of Slit and Robo genes in the developing mouse heart. *Dev Dyn*. 2010;239:3303-11.

46. Mommersteeg MT, Andrews WD, Ypsilanti AR, Zelina P, Yeh ML, Norden J, Kispert A, Chédotal A, Christoffels VM, Parnavelas JG. Slit-roundabout signaling regulates the development of the cardiac systemic venous return and pericardium. *Circ Res*. 2013;112:465-75.

47. Liu J, Zhang L, Wang D, Shen H, Jiang M, Mei P, Hayden PS, Sedor JR, Hu H. Congenital diaphragmatic hernia, kidney agenesis and cardiac defects associated with Slit3-deficiency in mice. *Mech Dev*. 2003;120:1059-70.

48. Kruszka P, Tanpaiboon P, Neas K, Crosby K, Berger SI, Martinez AF, Addissie YA, Pongprot Y, Sittiwangkul R, Silvilairat S, et al. Loss of function in ROBO1 is associated with tetralogy of Fallot and septal defects. *J Med Genet*. 2017;54:825-829.

49. Pinto D, Pagnamenta AT, Klei L, Anney R, Merico D, Regan R, Conroy J, Magalhaes TR, Correia C, Abrahams BS, et al. Functional impact of global rare copy number variation in autism spectrum disorders. *Nature*. 2010;466:368-72.

American Heart Association
Circulation: Genomic and Precision Medicine

Table 1. Number of cases in previous and current meta-analysis studies as well as controls used in the current study.

Databases for CHD cases	Thorsson *et al.* study	Current study	Databases for controls	Current study
**DECIPHER**	279	1,252	**1,000 Genome phase 3**	2,504
**ISCA**	331	1,107	**DGV**	>6,430
**Published literature**	814	1,900	**gnomAD**	10,738
**CHDwiki**	328	328	**Published literature**	356
**ECARUCA**	0	47	**WTCC2**	~6,000
**WES TOF (Page *et al.* 2019)**	N/A	829	**DDD**	845

DECIPHER= Database of Chromosomal Imbalance and Phenotype in Humans using Ensembl Resources, ISCA= International Standards for Cytogenomic Arrays, ECARUCA= European Cytogeneticists Association Register of Unbalanced Chromosome Aberrations, WES TOF= whole exome sequencing of Tetralogy of Fallot, DGV= Database of Genomic Variants, WTCC2= Wellcome Trust Case Control Consortium 2, DDD= Deciphering Developmental Disorders study

American Heart Association.
Circulation: Genomic and Precision Medicine

Table 2. Candidate genes supported by both CNV and WES data of CHD cases. 54 protein-coding candidate genes supported by CNV and WES data in non-syndromic CHD cases. All genes in the list are strict ohnologs. Data presented includes the Ensembl ID and the nature of the chromosomal imbalance for which the gene is either deleted (DEL), duplicated (DUP) or deleted/duplicated (BOTH).

ENS gene ID	Gene name	Chr	Start (hg19)	End (hg19)	DEL/DUP/BOTH	TOF LOF var count	TOF HIGH impact var count	TOF MED impact var count	Case CNVs overlap: FULL	Case CNVs overlap: PARTIAL
**ENSG00000058668**	ATP2B4	1	203595689	203713209	DUP	0	0	13	3	1
**ENSG00000064042**	LIMCH1	4	41361624	41702061	DUP	6	6	21	4	0
**ENSG00000083223**	ZCCHC6	9	88902648	88969369	DUP	1	1	8	4	0
**ENSG00000092847**	AGO1	1	36335409	36395211	DUP	0	0	2	3	0
**ENSG00000094916**	CBX5	12	54624724	54673886	DUP	0	0	1	2	0
**ENSG00000101367**	MAPRE1	20	31407699	31438211	DUP	0	0	1	5	0
**ENSG00000108387**	SEPT4	17	56597611	56618179	DUP	0	0	6	5	1
**ENSG00000108389**	MTMR4	17	56566898	56595266	DUP	0	0	9	10	0
**ENSG00000109332**	UBE2D3	4	103715540	103790053	DUP	2	2	0	3	0
**ENSG00000109685**	WHSC1	4	1873151	1983934	DUP	0	0	12	6	0
**ENSG00000112079**	STK38	6	36461669	36515247	DUP	0	0	3	5	1
**ENSG00000113108**	APBB3	5	139937853	139973337	DUP	1	2	8	8	0
**ENSG00000122515**	ZMIZ2	7	44788180	44809477	DUP	1	1	10	10	0
**ENSG00000138641**	HERC3	4	89442199	89629693	DUP	0	0	5	14	0
**ENSG00000138835**	RGS3	9	116207011	116360018	DUP	1	1	19	5	0
**ENSG00000140403**	DNAJA4	15	78556428	78574538	DUP	0	0	8	9	0
**ENSG00000140497**	SCAMP2	15	75136071	75165706	DUP	0	0	1	6	0
**ENSG00000145147**	SLIT2	4	20254883	20622184	DUP	0	0	14	3	0
**ENSG00000146463**	ZMYM4	1	35734568	35887659	DUP	0	0	12	26	1
**ENSG00000179361**	ARID3B	15	74833518	74890472	DUP	0	0	6	5	0
**ENSG00000185658**	BRWD1	21	40556102	40693485	DEL	5	5	16	10	1
**ENSG00000151240**	DIP2C	10	320130	735683	DEL	1	1	15	4	0
**ENSG00000158161**	EYA3	1	28296855	28415207	DEL	1	1	2	4	0
**ENSG00000106070**	GRB10	7	50657760	50861159	DEL	1	2	8	3	0

American Heart Association.
Circulation: Genomic and Precision Medicine

**ENSG00000154127**	UBASH3B	11	122526383	122685181	DEL	1	1	5	9	0
**ENSG00000092199**	HNRNPC	14	21677295	21737653	BOTH	1	1	2	6	0
**ENSG00000056586**	RC3H2	9	125606835	125667620	BOTH	1	1	8	3	1
**ENSG00000184347**	SLIT3	5	168088745	168728133	BOTH	2	2	21	3	0
**ENSG00000137076**	TLN1	9	35696945	35732392	BOTH	4	4	22	9	0
**ENSG00000010017**	RANBP9	6	13621730	13711796	BOTH	0	0	14	3	0
**ENSG00000020577**	SAMD4A	14	55033815	55260033	BOTH	0	0	11	8	0
**ENSG00000033800**	PIAS1	15	68346517	68483096	BOTH	0	0	4	7	1
**ENSG00000064726**	BTBD1	15	83685174	83736106	BOTH	1	1	7	5	0
**ENSG00000083312**	TNPO1	5	72112139	72212560	BOTH	0	0	4	5	0
**ENSG00000091009**	RBM27	5	145583113	145718814	BOTH	0	0	7	4	0
**ENSG00000099204**	ABLIM1	10	116190872	116444762	BOTH	3	3	14	12	0
**ENSG00000100320**	RBFOX2	22	36134783	36424473	BOTH	0	0	5	2	0
**ENSG00000100330**	MTMR3	22	30279144	30426855	BOTH	0	0	11	4	0
**ENSG00000100592**	DAAM1	14	59655364	59838123	BOTH	0	0	9	14	0
**ENSG00000113649**	TCERG1	5	145826874	145891524	BOTH	0	0	6	9	0
**ENSG00000116191**	RALGPS2	1	178694300	178889238	BOTH	0	0	1	2	0
**ENSG00000120899**	PTK2B	8	27168999	27316903	BOTH	0	0	8	4	0
**ENSG00000127022**	CANX	5	179105629	179157926	BOTH	0	0	10	2	0
**ENSG00000135074**	ADAM19	5	156822542	157002783	BOTH	2	2	10	7	1
**ENSG00000137573**	SULF1	8	70378859	70573150	BOTH	1	1	10	3	0
**ENSG00000137962**	ARHGAP29	1	94614544	94740624	BOTH	0	0	12	3	0
**ENSG00000138107**	ACTR1A	10	104238986	104262482	BOTH	0	0	2	2	1
**ENSG00000155506**	LARP1	5	154092462	154197167	BOTH	2	2	13	6	0
**ENSG00000166747**	AP1G1	16	71762913	71843104	BOTH	0	1	4	4	1
**ENSG00000166888**	STAT6	12	57489191	57525922	BOTH	0	0	7	5	0
**ENSG00000180340**	FZD2	17	42634925	42636907	BOTH	0	0	8	8	0
**ENSG00000180776**	ZDHHC20	13	21950263	22033509	BOTH	1	1	2	6	0
**ENSG00000196914**	ARHGEF12	11	120207787	120360645	BOTH	0	0	14	12	0
**ENSG00000213079**	SCAF8	6	155054459	155155192	BOTH	0	0	13	14	0

TOF= tetralogy of Fallot, var=variants, MED=medium impact

American Heart Association®
Circulation: Genomic and Precision Medicine

Table 3. Top pathways overrepresented in our 54 candidate genes.

Pathway tool	Pathway name	# Entities found	# Entities total	Entities ratio (%)
**Reactome**	Axon guidance	8	583	1.372212693
**Ingenuity pathway analysis**	Axon guidance signalling pathway	7	501	1.397206
**Reactome**	Signaling by Receptor Tyrosine Kinases	5	521	0.959692898
**Reactome**	Cellular responses to external stimuli	5	621	0.805152979
**Reactome**	Signaling by Interleukins	5	641	0.780031201
**Reactome**	Developmental Biology	8	1177	0.679694138
**Reactome**	Adaptive Immune System	6	998	0.601202405
**Reactome**	Cytokine Signaling in Immune system	6	1056	0.568181818
**Reactome**	Signal Transduction	15	3202	0.468457214
**Reactome**	Post-translational protein modification	7	1594	0.439146801
**Reactome**	Immune System	11	2662	0.41322314
**Reactome**	Metabolism of proteins	9	2354	0.382327952

# - number

Circulation: Genomic and Precision Medicine

Figure Legends:

Figure 1: Overall methodology. Flowchart showing the methodology used to identify novel genetic loci for non-syndromic CHD cases. Potential pathogenic variants were novel or rare SNVs (either absent from ExAC or with frequency of <0.01). Candidate genes identified at the end of the workflow were subsequently analysed for ohnolog status.

Figure 2: Intersection of CNV and WES data. Numbers of genes involved in the final stages of the workflow depicted in Figure 1 are shown. Genes with assigned phenotypes (circles with dashed line) were excluded from further analysis.

American Heart Association
Circulation: Genomic and Precision Medicine

Figure 3: Ohnologs are enriched in CHD cases. Graphs show the percentage of genes that are **A)** ohnologs **B)** small scale duplications (SSD) and **C)** singletons. Statistical significance was tested using two-tailed Chi-square test with Yates's correction, $p<0.05$ was considered statistically significant.

Figure 4: Filtering process using large-scale genomic data resources**.** Both graphs are in logarithmic scale and represent the consecutive filtering of the genes using the different metrics for **A)** deleted (DEL) and both CNV genes **B)** duplicated (DUP) and both CNV genes. There is approximately 70% reduction in the number of candidate genes when we apply the evolutionary duplication metric – ohnolog. Also, none of our candidates were present in the list of homozygous deleted genes (non-essential) from the Sudmant study as well as not present in the list of genes curated from the DDD study.

Figure 5: Genes in the top significant pathways and biological processes**.** *SLIT2* and *SLIT3* genes were supported by multiple lines of evidence.

American Heart Association.
Circulation: Genomic and Precision Medicine

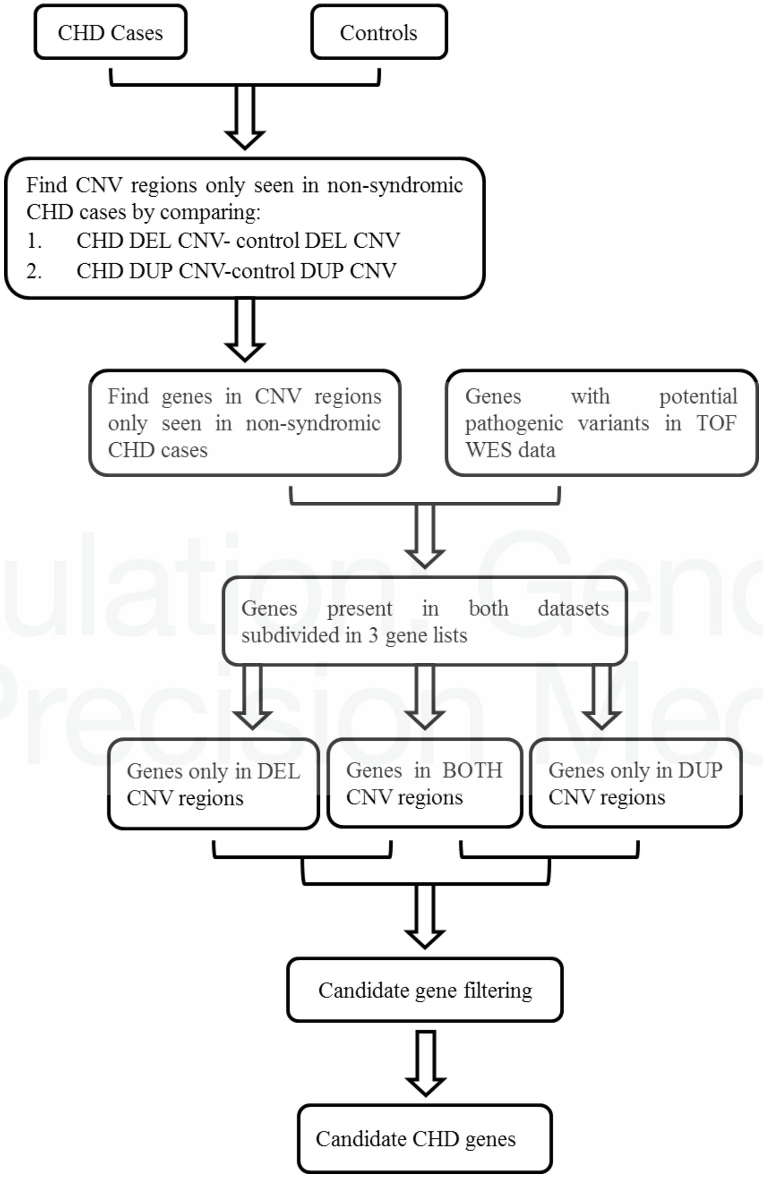

Circulation: Genomic and Precision Medicine
American Heart Association

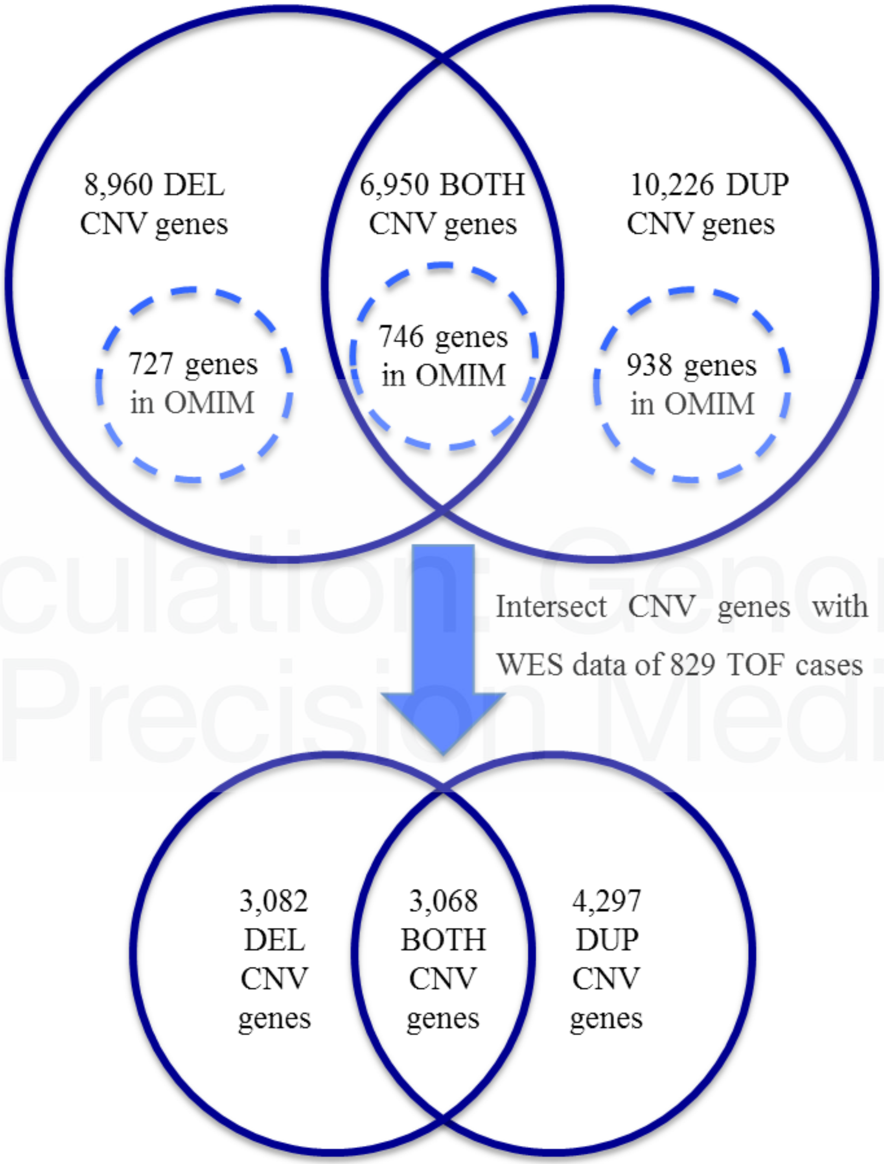

Circulation: Genomic and Precision Medicine
American Heart Association

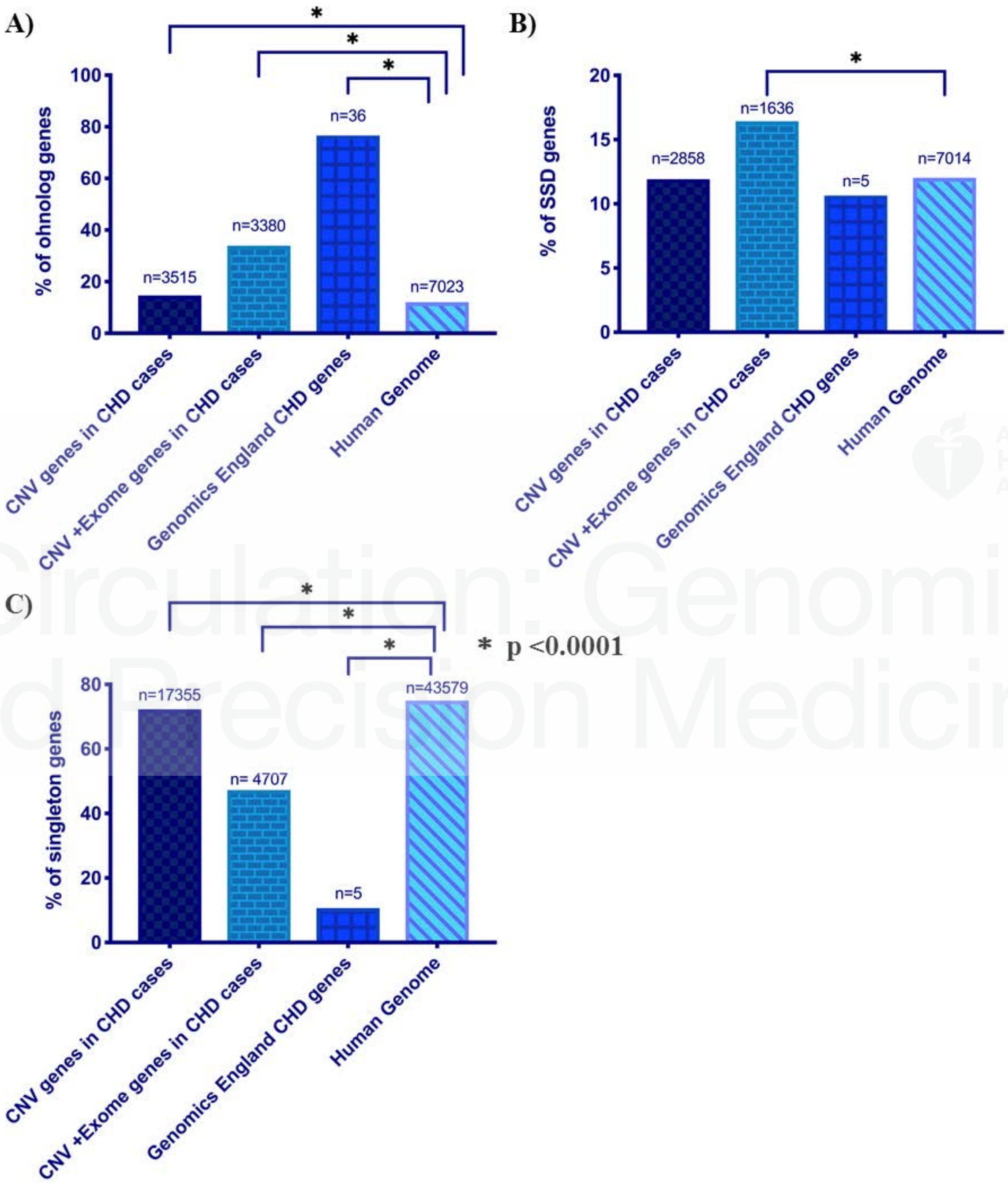

American Heart Association
Circulation: Genomic and Precision Medicine

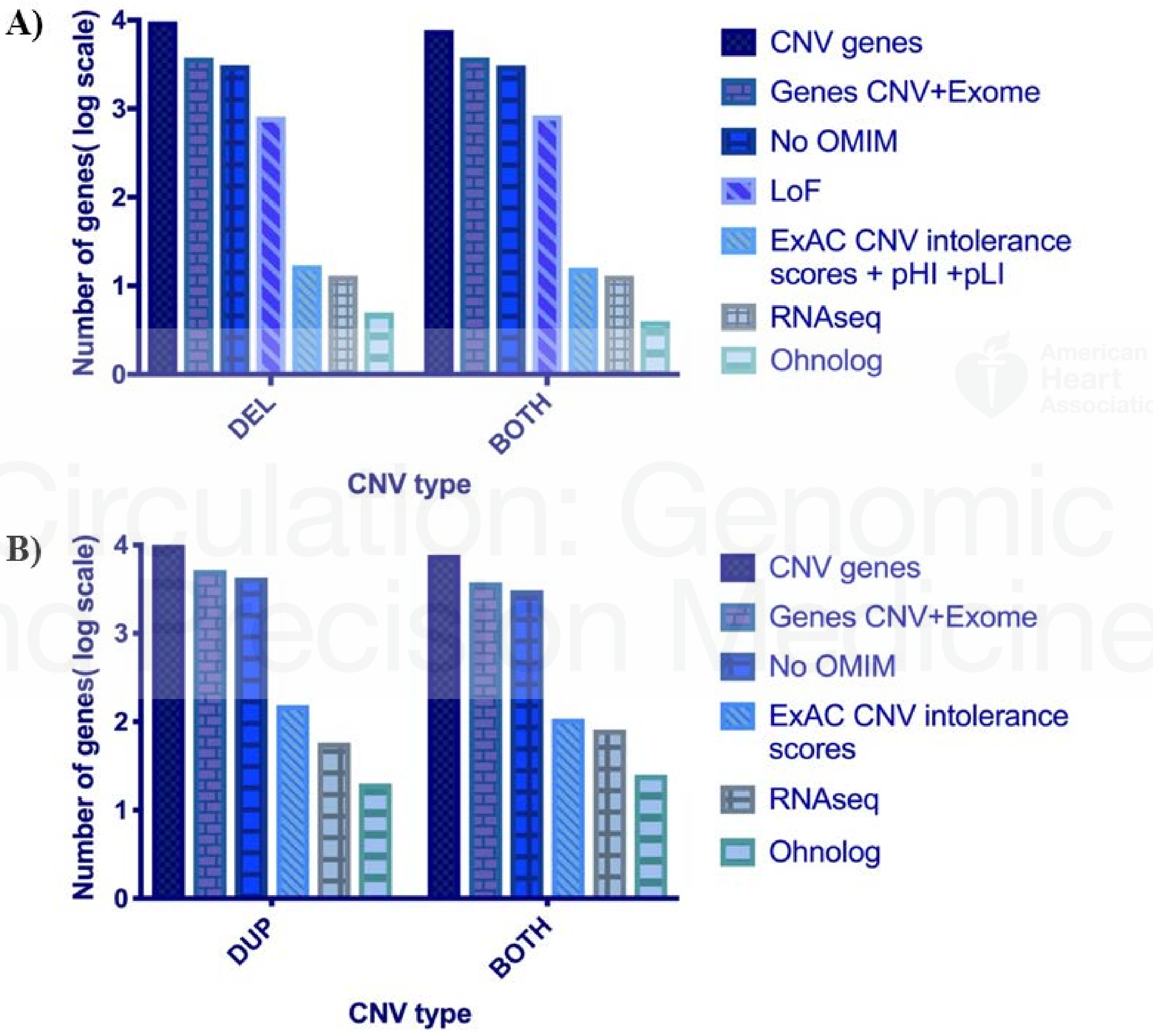

Circulation: Genomic and Precision Medicine
American Heart Association

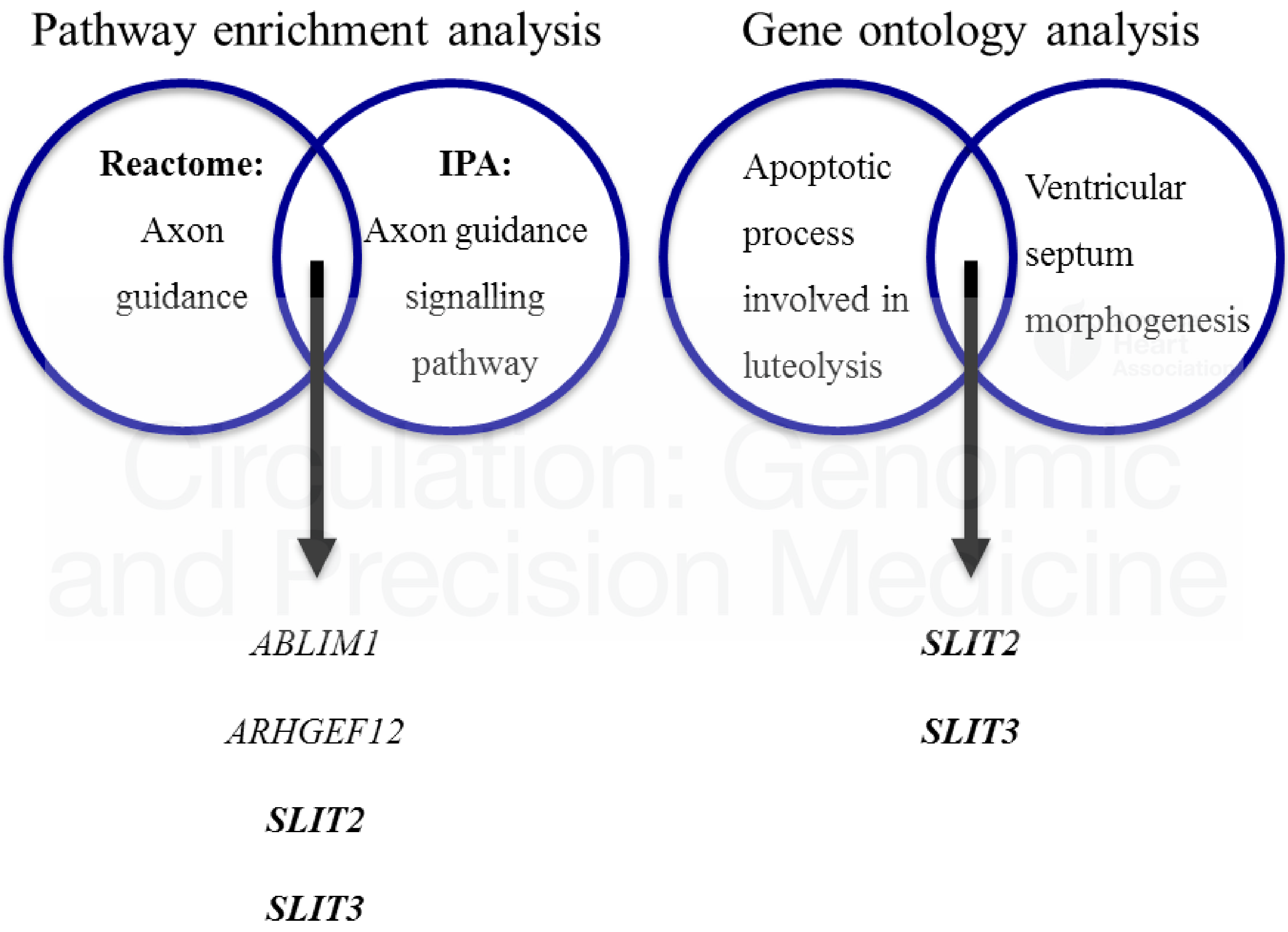

Circulation: Genomic and Precision Medicine
